# The predictive value of PD-L1 expression in response to anti-PD-1/PD-L1 therapy for biliary tract cancer: a systematic review and meta-analysis

**DOI:** 10.3389/fimmu.2024.1321813

**Published:** 2024-03-28

**Authors:** Seung Bae Yoon, Sang Myung Woo, Jung Won Chun, Dong Uk Kim, Jaihwan Kim, Joo Kyung Park, Hoonsub So, Moon Jae Chung, In Rae Cho, Jun Heo

**Affiliations:** ^1^ Department of Internal Medicine, Eunpyeong St. Mary’s Hospital, College of Medicine, The Catholic University of Korea, Seoul, Republic of Korea; ^2^ Center for Liver and Pancreatobiliary Cancer, National Cancer Center, Goyang, Republic of Korea; ^3^ Department of Internal Medicine, CHA University School of Medicine, Pocheon, Republic of Korea; ^4^ Department of Internal Medicine, Seoul National University Bundang Hospital, Seoul National University College of Medicine, Seongnam, Republic of Korea; ^5^ Department of Medicine, Samsung Medical Center, Sungkyunkwan University School of Medicine, Seoul, Republic of Korea; ^6^ Department of Internal Medicine, Ulsan University Hospital, University of Ulsan College of Medicine, Ulsan, Republic of Korea; ^7^ Department of Internal Medicine, Severance Hospital, Yonsei University College of Medicine, Seoul, Republic of Korea; ^8^ Department of Internal Medicine and Liver Research Institute, Seoul National University Hospital, Seoul National University College of Medicine, Seoul, Republic of Korea; ^9^ Department of Internal Medicine, Kyungpook National University School of Medicine, Daegu, Republic of Korea

**Keywords:** cholangiocarcinoma, programmed death ligand 1, immune checkpoint inhibitors, biomarker, immunohistochemistry

## Abstract

**Background:**

Recently, anti-programmed cell death protein 1 (PD-1)/programmed death-ligand 1 (PD-L1) immunotherapy offers promising results for advanced biliary tract cancer (BTC). However, patients show highly heterogeneous responses to treatment, and predictive biomarkers are lacking. We performed a systematic review and meta-analysis to assess the potential of PD-L1 expression as a biomarker for treatment response and survival in patients with BTC undergoing anti-PD-1/PD-L1 therapy.

**Methods:**

We conducted a comprehensive systematic literature search through June 2023, utilizing the PubMed, EMBASE, and Cochrane Library databases. The outcomes of interest included objective response rate (ORR), disease control rate (DCR), progression-free survival (PFS), and overall survival (OS) according to PD-L1 expression. Subgroup analyses and meta-regression were performed to identify possible sources of heterogeneity.

**Results:**

A total of 30 studies was included in the final analysis. Pooled analysis showed no significant differences in ORR (odds ratio [OR], 1.56; 95% confidence intervals [CIs], 0.94-2.56) and DCR (OR, 1.84; 95% CIs, 0.88-3.82) between PD-L1 (+) and PD-L1 (-) patients. In contrast, survival analysis showed improved PFS (hazard ratio [HR], 0.54, 95% CIs, 0.41-0.71) and OS (HR, 0.58; 95% CI, 0.47-0.72) among PD-L1 (+) patients compared to PD-L1 (-) patients. Sensitivity analysis excluding retrospective studies showed no significant differences with the primary results. Furthermore, meta-regression demonstrated that drug target (PD-1 *vs.* PD-L1), presence of additional intervention (monotherapy *vs.* combination therapy), and PD-L1 cut-off level (1% *vs.* ≥5%) significantly affected the predictive value of PD-L1 expression.

**Conclusion:**

PD-L1 expression might be a helpful biomarker for predicting PFS and OS in patients with BTC undergoing anti-PD-1/PD-L1 therapy. The predictive value of PD-L1 expression can be significantly influenced by diagnostic or treatment variables.

**Systematic review registration:**

https://www.crd.york.ac.uk/PROSPERO, identifier CRD42023434114.

## 1 Introduction

Biliary tract cancer (BTC) refers to a diverse group of malignant tumors arising from the biliary or gallbladder epithelium, including intrahepatic cholangiocarcinoma, extrahepatic cholangiocarcinoma, and gallbladder cancer. This disease has a poor prognosis, with a 5-year survival rate of <20% and increasing global mortality rates (1, 2). Surgery is the only curative treatment for BTC, although a minority are potential candidates for radial resection due to delayed diagnosis from a frequent lack of symptoms. Accordingly, palliative chemotherapy continues to be the mainstay of treatment for most patients with BTC, with gemcitabine plus cisplatin remaining the standard first-line therapy for more than a decade (3, 4). However, the limited median survival benefit of <1 year despite undergoing standard systemic chemotherapy highlights the need for more effective medical treatments.

The emergence of immunotherapy has revolutionized cancer treatment. In particular, immune checkpoint inhibitors (ICIs) that target the programmed cell death protein 1 (PD-1) or programmed death-ligand 1 (PD-L1) have shown promising outcomes for diverse solid tumors, including melanoma, non-small cell lung cancer, renal cell carcinoma, and bladder cancer (5–8). In BTC, immunotherapy using pembrolizumab was first evaluated in two cohorts of metastatic patients where cancer progressed after standard systemic chemotherapy: Keynote-158 (104 patients) and Keynote-028 (23 patients) (9). In these two cohorts, the objective response rate (ORR) with median progression-free survival (PFS) were 5.8% with 2.0 months and 13.0% with 1.8 months, respectively. Although ICIs have had considerable success in immunotherapy for some malignancies, most patients with BTC fail to achieve durable responses with ICI monotherapy. Therefore, it is crucial to develop effective combination regimens and explore potential biomarkers to identify patients who would benefit from immunotherapy. Furthermore, while recent phase 3 trials have shown promising outcomes with anti-PD-1/PD-L1 plus gemcitabine and cisplatin regimens (10, 11), a biomarker in patients with BTC undergoing immunotherapy remains to be established.

Considering the underlying mechanism of PD-1/PD-L1 inhibitor therapy, PD-L1 expression on tumor or immune cells serves as a promising biomarker for predicting ICI response. Immunohistochemistry (IHC) of PD-L1 is the most widely validated method for selecting patients for ICI therapy, demonstrating robust predictive values in various cancers (12–14). Recent meta-analyses have demonstrated the utility of PD-L1 expression as a valuable predictive biomarker of anti-PD-1/PD-L1 therapy in digestive malignancies, including gastroesophageal and hepatocellular carcinoma (15–17). However, the value of PD-L1 expression in patients with BTC receiving anti-PD-1/PD-L1 therapy remains controversial. Results from the initial Keynote-158 cohort reported no differences in ORR, PFS, or OS between PD-L1 (+) and PD-L1 (-) patients treated with pembrolizumab (9). Conversely, in a phase 2 multi-institutional study of nivolumab for patients with advanced BTC, positive PD-L1 expression was associated with prolonged PFS (18). In addition to these conflicting results, the definition of PD-L1 positivity remains unclear, resulting in the use of several PD-L1 scoring methods and cut-offs in clinical trials. Moreover, no optimal anti-PD-1/PD-L1 drug or combination immunotherapy regimen has been identified for BTC.

Given these circumstances, the aim of this meta-analysis and systematic review was to assess the value of PD-L1 expression for predicting tumor response and survival outcomes with PD-1/PD-L1 inhibitors in patients with BTC. We also aimed to identify variables associated with the predictive performance of PD-L1 expression in this cohort.

## 2 Materials and methods

This systematic review and meta-analysis has been registered in PROSPERO (CRD42023434114) and was conducted according to the Preferred Reporting Items for Systematic Reviews and Meta-Analyses guidelines (**Supplementary Table 1**) (19).

### 2.1 Data sources and search strategy

A comprehensive systematic literature search through June 20, 2023, utilizing the PubMed, EMBASE, and Cochrane Library databases. The key search terms included “cholangiocarcinoma,” “biliary tract neoplasms,” “gallbladder neoplasms,” “Immune checkpoint inhibitors,” “PD-1,” and “PD-L1.” The full search details are presented in **Supplementary Table 2**. Included studies were restricted to English publications. Additional relevant studies by manually cross-checking the reference lists in the retrieved articles.

### 2.2 Study selection

All randomized trials and prospective/retrospective studies fulfilling the following criteria were included: [1] patients with BTC treated with PD-1 or PD-L1 inhibitors; [2] PD-L1 status based on IHC staining methods; and [3] presentation of at least one of the clinical outcomes of ORR, disease control rate (DCR), PFS, or OS. The exclusion criteria were as follows: [1] the inclusion of only PD-L1 (+) patients; [2] lacking or no data on ORR, DCR, PFS, or OS; and [3] non-English publications, case series (n<10), letters, commentaries, or review papers. In cases where multiple studies reported data from the same cohort, the higher quality study was included. Two investigators (SBY and SMW) independently reviewed and evaluated all titles, abstracts, and full texts. Discrepancies between the two investigators were resolved through discussion with a third reviewer (JWC).

### 2.3 Data extraction and quality assessment

Data were extracted independently by two investigators (SBY and SMW) using a standardized table. The following items were documented for each study: [1] study characteristics, including the name of the first author, publication year, study design, region, and median follow-up period; [2] participant characteristics, including number of patients, median age, and sex; [3] intervention details, including name of drug, line of therapy, and combined treatment; [4] PD-L1 expression assessment, including type of PD-L1 antibody clone, PD-L1 IHC scoring method, and respective cut-off values; and [5] clinical outcomes (ORR, DCR, median PFS, and median OS) and safety (grade 3-5 adverse event rates) profiles. For studies with multiple combination drug modalities including anti-PD-1/PD-L1 therapy, data were collected separately and analyzed as individual datasets. If the hazard ratio (HR) of survival data was not provided in the original paper, the values were extracted by replicating Kaplan-Meier survival curves using WebPlotDigitizer software Version 4.5 (PLOTCON; Oakland, CA, USA). As a meta-analysis of biomarker assessment, the Quality in Prognosis Studies (QUIPS) tool was used to appraise the quality of the included studies based on six domains: [1] study participants, [2] study attrition, [3] prognostic factor measurement, [4] outcome measurement, [5] adjustment for other prognostic factors, and [6] statistical analysis and reporting (20, 21).

### 2.4 Data synthesis and statistical analysis

Pooled ORR and DCR result were derived using the random-effects model, as suggested by DerSimonian and Laird (22). Time-to-event (PFS and OS) data were incorporated into the meta-analysis, according to the methodology employed by Tierney et al. (23). Forest plots were constructed for the visual representation of individual study results and pooled data. Sensitivity analyses were performed for clinical trials and prospective studies, excluding retrospective studies. Meanwhile, subgroup analyses and meta-regression were performed according to region (Eastern *vs.* Western), target of drug (PD-1 *vs.* PD-L1), line of therapy (1^st^ *vs.* 2^nd^ or later), presence of additional interventions (monotherapy *vs.* combination therapy), PD-L1 scoring method (tumor proportion score [TPS] *vs.* combined positive score [CPS]), and PD-L1 cut-off level (1% *vs.* ≥5%). Furthermore, publication bias was qualitatively evaluated by visual inspection of the funnel plot and statistically confirmed using Egger’s test. This meta-analysis was conducted using the Comprehensive Meta-Analysis Software Version 4.0 (Biostat, Englewood, NJ, USA).

## 3 Results

### 3.1 Search results and population characteristics

**Figure 1:**
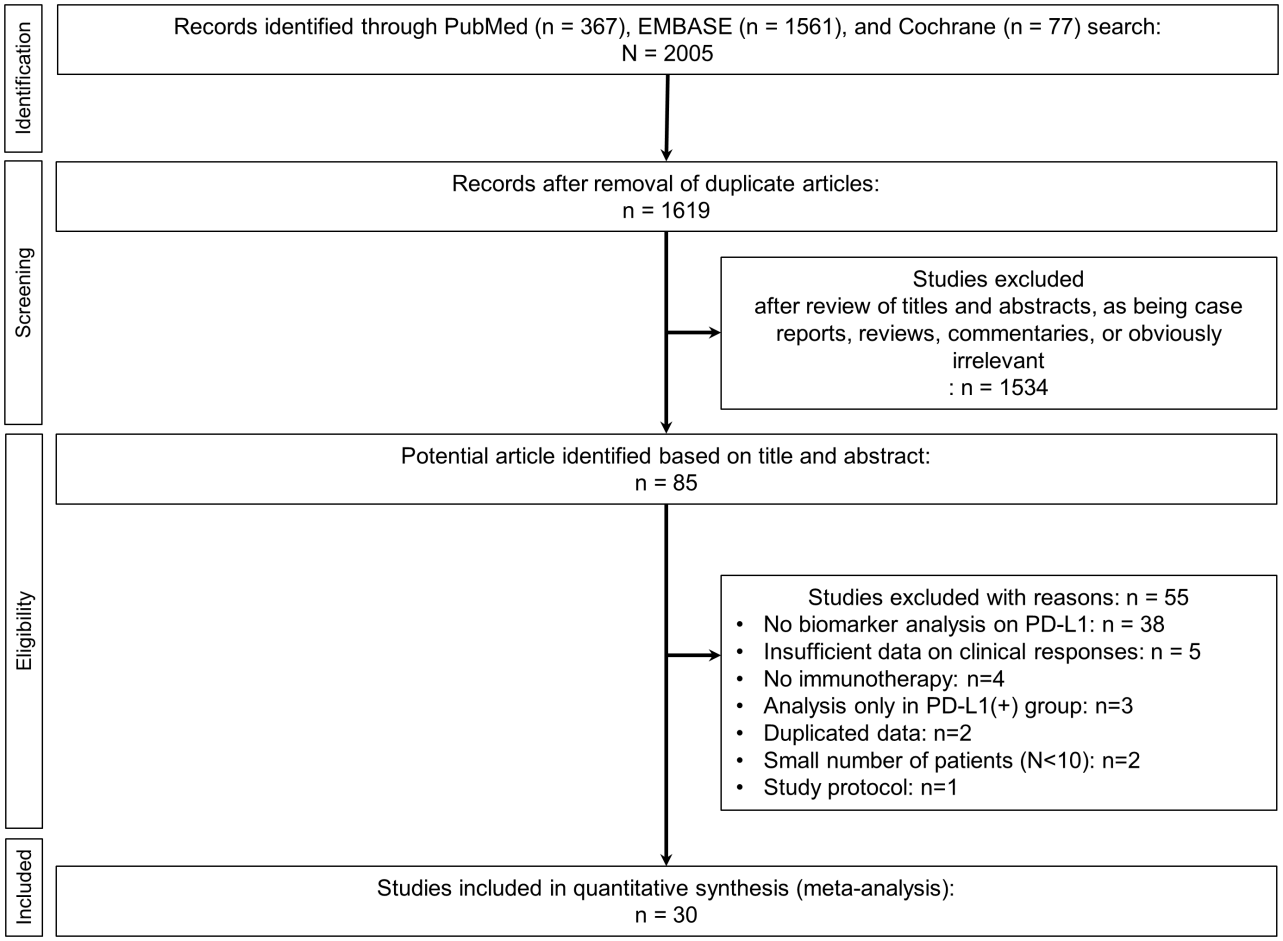
Study flow chart.

The initial literature search retrieved 2005 articles. After removing duplicates, 1619 remained for further title and abstract review (**Figure 1**). After excluding case reports, reviews, commentaries, and irrelevant articles, 85 studies underwent full analysis. Of these 85 studies, 55 were excluded due to the following reasons: [1] lack of biomarker analysis for PD-L1 (n=38), [2] insufficient data on clinical responses (n=5), [3] non-use of anti-PD-1/PD-1 therapy (n=4), [4] analysis only in PD-L1 (+) patients (n=3), [5] duplicated data (n=2), [6] small number (N<10) of patients (n=2), and (7) discussion of study protocol only (n=1). Ultimately, a total of 30 studies was included in final analysis (9, 18, 24–51).

### 3.2 Characteristics and quality of the included studies

Of the 30 studies, 18 were phase I/II clinical trials, two were prospective studies, and the remaining 10 were retrospective studies. Most studies (83.3%, 25/30) were conducted in Asian countries. PD-1 inhibitors, including pembrolizumab, nivolumab, camrelizumab, sintilimab, toripalimab, and tislelizumab, were used in 24 studies, whereas PD-L1 inhibitors, including durvalumab, avelumab, and bintrafusp alfa, were used in six studies. Since three studies included two or more intervention modalities (26, 37, 41), a total of 34 datasets with 1310 patients were included in the meta-analysis. Among these patients, 631 (48.2%) were PD-L1 (+). Main characteristics of the included studies are summarized in **Table 1**.

**Table 1:** Characteristics of included studies.

Study	Design	Region	Drug name (Target)	Additional treatment	Line of therapy	PD-L1 antibody clone	Definition of PD-L1 positivity	PD-L1 expression, n (+/-)
Arkenau et al., 2018 (24)	Phase 1 trial	European multinational	Pembrolizumab (PD-1)	None	≥ 2nd	22C3	CPS ≥1	12/12
Gou et al., 2019 (25)	Retrospective study	China	Nivolumab (PD-1)	None or with various chemotherapies	≥ 1st	NR	TPS ≥1	11/19
Ueno et al., 2019 (26) (sub1)	Phase 1 trial	Japan	Nivolumab (PD-1)	None	≥ 2nd	28–8	CPS ≥1	18/10
Ueno et al., 2019 (26) (sub2)	Phase 1 trial	Japan	Nivolumab (PD-1)	GemCis	1st	28–8	CPS ≥1	18/10
Chen et al., 2020 (27)	Phase 2 trial	China	Camrelizumab (PD-1)	GEMOX	1st	NR	TPS ≥1	5/26
Feng et al., 2020 (28)	Phase 2 trial	China	Nivolumab (PD-1)	GemCis	≥ 1st	22C3	TPS ≥1	10/11
Kang et al., 2020 (29)	Prospective study	Korea	Pembrolizumab (PD-1)	None	≥ 2nd	22C3	TPS ≥1	31/8
Kim et al., 2020 (18)	Phase 2 trial	USA	Nivolumab (PD-1)	None	≥ 2nd	NR	TPS ≥1	18/23
Lin et al., 2020 (30)	Phase 2b trial	China	Pembrolizumab (PD-1)	Lenvatinib	≥ 2nd	22C3	TPS ≥5	11/21
Piha-Paul et al., 2020 (9)	Phase 2 trial	Multinational	Pembrolizumab (PD-1)	None	≥ 2nd	22C3	CPS ≥1	61/35
Yoo et al., 2020 (31)	Phase 1 trial	Asian Multinational	Bintrafusp alfa (PD-L1&TGF-β)	None	≥ 2nd	73-10	TPS ≥1	14/12
Wang et al., 2021 (32)	Phase 2 trial	China	Camrelizumab (PD-1)	Apatinib	≥ 2nd	22C3	CPS ≥1	4/17
Zhang et al., 2021 (33)	Phase 2 trial	China	Various (PD-1)	Lenvatinib	1st	22C3	CPS ≥1	18/11
Chiang et al., 2022 (34)	Phase 2 trial	Taiwan	Nivolumab (PD-1)	Gemcitabine+S1	1st	22C3	CPS ≥1	20/27
Cousin et al., 2022 (35)	Phase 2 trial	France	Avelumab (PD-L1)	Regorafenib	≥ 2nd	QR1	High vs. Low	13/14
Ding et al., 2022 (36)	Retrospective study	China	Sintilimab (PD-1)	Lenvatinib	2nd	22C3	TPS ≥1	32/9
Doki et al., 2022 (37) (sub1)	Phase 1 trial	Asian multinational	Durvalumab (PD-L1)	None	≥ 2nd	SP263	TPS ≥1	19/18
Doki et al., 2022 (37) (sub2)	Phase 1 trial	Asian multinational	Durvalumab (PD-L1)	Tremelimumab	≥ 2nd	SP263	TPS ≥1	18/35
Dong et al., 2022 (38)	Prospective study	China	Not specified	GEMOX+Lenvatinib	1st	22C3 or 28-8	TPS ≥1	4/8
Kim et al., 2022 (39)	Retrospective study	Korea	Pembrolizumab or nivolumab (PD-1)	None	≥ 2nd	22C3	CPS ≥1	56/27
Li et al., 2022 (40)	Phase 2 trial	China	Toripalimab (PD-1)	Gemcitabine and S-1	1st	22C3	CPS ≥1	16/16
Oh et al., 2022 (41)	Phase 2 trial	Korea	Durvalumab (PD-L1)	GemCis+Tremelimumab (biomarker cohort)	1st	SP263	TC ≥1 or IC ≥1	18/10
Oh et al., 2022 (41)	Phase 2 trial	Korea	Durvalumab (PD-L1)	GemCis+Tremelimumab	1st	SP263	TC ≥1 or IC ≥1	29/16
Oh et al., 2022 (41)	Phase 2 trial	Korea	Durvalumab (PD-L1)	GemCis	1st	SP263	TC ≥1 or IC ≥1	30/15
Shi et al., 2022 (42)	Retrospective study	China	Various (PD-1)	Lenvatinib	≥ 2nd	E1L3N	CPS ≥50	8/14
Tan et al., 2022 (43)	Retrospective study	China	Various (PD-1)	Nab-paclitaxel-containing chemotherapy	2nd	NR	Not specified	1/6
Zuo et al., 2022 (44)	Retrospective study	China	Various (PD-1)	Lenvatinib	≥ 2nd	NR	NR	13/18
Jeong et al., 2023 (45)	Retrospective study	Korea	Pembrolizumab or nivolumab (PD-1)	None	≥ 2nd	22C3	TPS ≥1	21/27
Jin et al., 2023 (46)	Phase 2 trial	China	Sintilimab (PD-1)	Anlotinib	2nd	22C3	CPS ≥10	6/14
Shi et al., 2023 (47)	Phase 2 trial	China	Toripalimab (PD-1)	GEMOX+Lenvatinib	1st	SP142	CPS ≥1	14/16
Wang et al., 2023 (48)	Retrospective study	China	Toripalimab (PD-1)	Lenvatinib ± Locoregional therapies	≥ 2nd	E1L3N	CPS ≥1	14/20
Yoo et al., 2023 (49)	Phase 2 trial	Multinational	Bintrafusp alfa (PD-L1&TGF-β)	None	2nd	SP263	TPS ≥1	43/98
Zhu et al., 2023 (50)	Retrospective study	China	Various (PD-1)	GEMOX+Lenvatinib	1st	NR	CPS ≥5	17/28
Zhu et al., 2023 (50)	Retrospective study	China	Various (PD-1/PD-L1)	GEMOX+Lenvatinib	≥ 1st	NR	CPS ≥5	8/28

CPS, Combined positive score; IC, Immune cells; ICI, immune checkpoint inhibitors; GemCis, Gemcitabine+Cisplatin; GEMOX, Gemcitabine+Oxaliplatin; NR, not reported; TC, Tumor cells; TPS, Tumor proportion score.

The QUIPS tools, which assessed the risk of bias in the included studies, showed that 63% (19/30) of the studies had at least one domain at high risk of bias. Reasons for high risk of bias included the following: [1] retrospective design (n=10), [2] varied ICIs and treatment modalities (n=7), [3] insufficient reporting data on clinical outcomes (n=7). and [4] inconsistency of PD-L1 IHC methods or clones (n=4). Further details of the quality assessment are presented in **Supplementary Table 3**.

### 3.3 Clinical outcomes of all patients

Pooled analysis revealed an ORR of 28.8% (95% confidence intervals [CIs], 23.8–34.3%; *I* ^2^ = 87.4%) and a DCR of 68.6% (95% CIs, 62.6–74.0%; *I* ^2^ = 90.4%). The pooled rate of grade 3-5 adverse events was 36.7% (95% CIs, 30.1–43.8%; *I* ^2^ = 84.8%). Compared to the ICI-monotherapy group, pooled ORR (9.0%; 95% CIs, 5.6–14.2%; *I* ^2^ = 5.3% *vs.* 40.2%; 95% CIs, 33.2–47.6%; *I* ^2^ = 80.0%; *P*<0.001), DCR (36.4%; 95% CIs, 26.9–47.1%; *I* ^2^ = 77.7% *vs.* 83.0%; 95% CIs, 77.7–87.3%; *I* ^2^ = 68.8%; *P*<0.001), and grade 3-5 adverse event rates (20.7%; 95% CIs, 13.2–30.9%; *I* ^2^ = 73.9% *vs.* 47.4%; 95% CIs, 38.5–56.4%; *I* ^2^ = 78.0%; *P*<0.001) were significantly higher in the ICI-based combination therapy group. The detailed clinical outcomes of all patients from the included studies are summarized in **Supplementary Table 4**. The forest plots of these clinical outcomes are presented in **Supplementary Figure 1**.

### 3.4 Clinical outcomes according to PD-L1 expression

**Figure 2:**
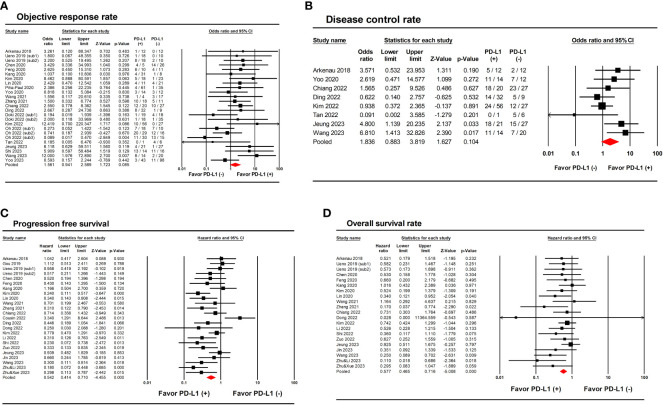
Forest plots showing the results of primary outcomes in patients with biliary tract cancer treated with anti PD-1/PD-L1 therapy according to PD-L1 expression. **(A)** Objective response rate. **(B)** Disease control rate. **(C)** Progression-free survival. **(D)** Overall survival.

Primary clinical outcomes according to PD-L1 expression are presented in **Table 2**. The ORR, DCR, PFS, and OS according to PD-L1 expression were compared between PD-L1 (+) and PD-L1 (-) groups. Overall, 25 (n=1055) and 8 (n=310) studies reported ORR and DCR according to PD-L1 expression, respectively. A direct comparison between PD-L1 (+) and PD-L1 (-) groups showed no significant differences in ORR (odds ratio [OR], 1.56; 95% CIs, 0.94-2.56; *I* ^2^ = 37.7%; *P*=0.085) and DCR (OR, 1.84; 95% CIs, 0.88-3.82; *I* ^2^ = 39.7%; *P*=0.104) (**Figures 2A, B**). On the other hand, 24 (n=802) and 21 (n=704) studies reported PFS and OS according to PD-L1 expression, respectively. Pooled analysis showed significantly improved PFS (HR, 0.54; 95% CIs, 0.41-0.71; *I* ^2^ = 54.2%; *P*<0.001) and OS (HR, 0.58; 95% CIs, 0.47-0.72; *I* ^2^ = 0.0%; *P*<0.001) in PD-L1 (+) compared to PD-L1 (-) patients (**Figures 2C, D**).

**Table 2:** Clinical outcomes according to PD-L1 expression.

Study	PD-L1 positive					PD-L1 negative				
	n (%)	ORR, %	DCR, %	mPFS (95% CI), months	mOS (95% CI), months	n (%)	ORR, %	DCR, %	mPFS (95% CI), months	mOS (95% CI), months
Arkenau et al., 2018 (24)	12 (50)	8.3	41.7	1.5 (1.2-4.2)	1.6 (1.3-2.1)	12 (50)	0.0	16.7	1.6 (1.3-2.1)	6.1 (3.5-8.3)
Gou et al., 2019 (25)	11 (36.7)	NR	NR	3.6 (0.7-6.5)	NR	19 (63.3)	NR	NR	3.0 (1.9-4.2)	NR
Ueno et al., 2019 (26) (sub1)	18 (64.3)	5.6	NR	1.4 (1.4-1.5)	8.7 (4.6-11.6)	10 (35.7)	0.0	NR	1.4 (1.1-3.4)	4.3 (3.1-6.4)
Ueno et al., 2019 (26) (sub2)	18 (64.3)	44.4	NR	4.3 (2.8-7.9)	NR (11.8-NR)	10 (35.7)	20.0	NR	4.1 (1.4-4.4)	15.4 (5.7-15.4)
Chen et al., 2020 (27)	5 (16.1)	80.0	NR	9 (8.5-NE)	17.8 (14.2-NE)	26 (83.9)	53.8	NR	6.0 (4.2-7.1)	11.9 (8.4-21.9)
Feng et al., 2020 (28)	10 (47.6)	60.0	NR	6.3 (1.7-NE)	8.6 (1.9-NE)	11 (52.4)	36.4	NR	4.3 (3.0-7.9)	12.5 (4.7-NE)
Kang et al., 2020 (29)	31 (79.5)	12.9	NR	2.4 (1.0-3.8)	4.3 (3.2-5.5)	8 (20.5)	12.5	NR	2.8 (2.6-3.0)	4.8 (2.4-7.1)
Kim et al., 2020 (18)	18 (43.9)	27.8	NR	10.4 (NE)	Not reached	23 (56.1)	4.3	NR	2.3 (2.1-2.7)	10.8 (4.7-16.9)
Lin et al., 2020 (30)	11 (34.4)	36.4	NR	6.3 (NR)	20.7 (NR)	21 (65.6)	19.0	NR	8.4 (NR)	20.7 (NR)
Piha-Paul et al., 2020 (9)	61 (63.5)	6.6	NR	1.9 (1.8-2.0)	7.2 (3.7-10.8)	35 (36.5)	2.9	NR	2.1 (1.9-2.6)	9.3 (4.2-11.5)
Yoo et al., 2020 (31)	14 (53.8)	21.4	78.6	NR	NR	12 (46.2)	25.0	58.3	NR	NR
Wang et al., 2021 (32)	4 (19)	25.0	NR	5.6 (0.9-10.2)	10.6 (0.0-19.4)	17 (81)	17.6	NR	4.4 (2.51-6.34)	13.1 (8.1-18.2)
Zhang et al., 2021 (33)	18 (62.1)	55.6	NR	13.4 (NR)	Not reached	11 (37.9)	45.5	NR	13.4 (NR)	6.4 (NR)
Chiang et al., 2022 (34)	20 (42.6)	60.0	90.0	NR	NR	27 (57.4)	37.0	85.2	NR	NR
Cousin et al., 2022 (35)	13 (48.1)	NR	NR	NR	NR	14 (51.9)	NR	NR	NR	NR
Ding et al., 2022 (36)	32 (78)	25.0	43.8	7.6 (5.2-10.0)	16.6 (5.8-27.4)	9 (22)	11.1	55.6	3.8 (1.5-6.1)	5 (0.0-19.4)
Doki et al., 2022 (37) (sub1)	19 (51.4)	5.3	NR	NR	NR	18 (48.6)	22.2	NR	NR	NR
Doki et al., 2022 (37) (sub2)	18 (34)	5.6	NR	NR	NR	35 (66)	2.9	NR	NR	NR
Dong et al., 2022 (38)	4 (33.3)	25.0	25.0	9.7 (NE)	Not reached	8 (66.7)	NR	NR	4.8 (0.2-9.5)	Not reached
Kim et al., 2022 (39)	56 (67.5)	17.9	42.9	2.9 (2.4-3.4)	8.1 (6.0-10.3)	27 (32.5)	0.0	44.4	2.6 (2.3-2.9)	6.3 (5.4-7.2)
Li et al., 2022 (40)	16 (50)	6.3	NR	14.5 (NR)	16.1 (NR)	16 (50)	NR	NR	4.85 (NR)	12 (NR)
Oh 2022 (sub1)	18 (64.3)	38.9	NR	17.7 (3.4-32.0)	NR	10 (35.7)	70.0	NR	12.8 (11.1-14.5)	NR
Oh 2022 (sub2)	29 (64.4)	69.0	NR	11.7 (9.6-13.7)	NR	16 (35.6)	75.0	NR	16.8 (7.4-26.1)	NR
Oh 2022 (sub3)	30 (66.7)	36.7	NR	11.7 (9.6-13.7)	NR	15 (33.3)	86.7	NR	16.8 (7.4-26.1)	NR
Shi et al., 2022 (42)	8 (36.4)	NR	NR	6.5 (4.0-8.0)	13 (9.5-NE)	14 (63.6)	NR	NR	4.0 (2.0-5.0)	8.5 (6.5-12.0)
Tan et al., 2022 (43)	1 (14.3)	0.0	0.0	NR	NR	6 (85.7)	66.7	83.3	NR	NR
Zuo et al., 2022 (44)	13 (41.9)	NR	NR	7.1 (NR)	20.7 (NR)	18 (58.1)	NR	NR	4.2 (NR)	8 (NR)
Jeong et al., 2023 (45)	21 (43.8)	19.0	85.7	3.6 (2.8-4.4)	6.1 (3.0-9.2)	27 (56.3)	3.7	55.6	4.1 (3.0-5.3)	5.2 (3.1-7.3)
Jin et al., 2023 (46)	6 (30)	NR	NR	7.4 (4.9-9.9)	22.5 (10.0-35.2)	14 (70)	NR	NR	3.5 (0.0-8.7)	12.4 (10.2-14.5)
Shi et al., 2023 (47)	14 (46.7)	92.9	NR	NR	NR	16 (53.3)	68.8	NR	NR	NR
Wang et al., 2023 (48)	14 (41.2)	57.1	78.6	12.7 (0-25.4)	16.2 (10.7-21.6)	20 (58.8)	10.0	35.0	5.9 (3.0-8.8)	8.4 (2.0-14.9)
Yoo et al., 2023 (49)	43 (30.5)	7.0	NR	NR	NR	98 (69.5)	11.2	NR	NR	NR
Zhu et al., 2023 (50)	17 (37.8)	NR	NR	13.2 (NR)	Not reached	28 (62.2)	NR	NR	6.9 (NR)	9.6 (NR)
Zhu et al., 2023 (50)	8 (22.2)	NR	NR	12.0 (10.3-13.7)	21.4 (NE)	28 (77.8)	NR	NR	7.1 (3.7-10.5)	11.6 (6.9-16.4)

CI, confidence intervals, DCR, disease control rate; mPFS, median progression-free survival; mOS, median overall survival; NE, not estimable; NR, not reported; ORR, objective response rate.

### 3.5 Sensitivity analysis

Sensitivity analysis was performed only for clinical trials and prospective studies. This analysis revealed that PD-L1 (+) expression did not significantly improve ORR (OR, 1.25; 95% CIs, 0.75-2.09; *I* ^2^ = 30.4%; *P*=0.387) and DCR (OR, 2.42; 95% CIs, 0.86-6.86; *I* ^2^ = 0.0%; *P*=0.096) between groups (**Supplementary Figures 2A, B**). In contrast, sensitivity analysis showed that PD-L1 (+) expression was associated with improved PFS (HR, 0.60; 95% CIs, 0.42-0.86; *I* ^2^ = 53.6%; *P=*0.005) and OS (HR, 0.59; 95% CIs, 0.43-0.76; *I* ^2^ = 0.0%; *P*<0.001) (**Supplementary Figures 2C, D**). This confirms the robustness of the findings since sensitivity analysis did not significantly change the primary results.

### 3.6 Subgroup analysis and meta-regression

Subgroup analyses and meta-regression were performed for ORR, PFS, and OS using six variables (**Table 3**). The predictive value of PD-L1 expression was significantly affected by drug target (PD-1 *vs.* PD-L1), presence of additional interventions (monotherapy *vs.* combination therapy), and PD-L1 cut-off level (1% *vs.* ≥5%). Specifically, use of anti-PD-1 inhibitors was associated with higher OR of ORR (2.97; 95% CIs, 1.84–4.79 *vs.* 0.42; 95% CIs, 0.22-0.81; *P*<0.001) and lower HR of PFS (0.53; 95% CIs, 0.41-0.67 *vs.* 3.34; 95% CIs, 1.01-11.01; *P*=0.003) compared to use of anti-PD-L1 inhibitors. Studies with ICI-based combination therapy showed lower HR of OS (0.45; 95% CIs, 0.34-0.61 *vs.* 0.75; 95% CIs, 0.55-1.02; *P*=0.019) than studies with ICI-monotherapy. Furthermore, higher PD-L1 cut-off values (≥5%) were associated with lower HR of PFS (0.31; 95% CIs, 0.18-0.54 *vs.* 0.59; 95% CIs, 0.44-0.79; *P*=0.017) and lower HR of OS (0.30; 95% CIs, 0.17-0.53 *vs.* 0.65; 95% CIs, 0.51-0.82; *P*=0.015) compared to lower PD-L1 cut-off values (1%).

**Table 3:** Subgroup analyses and meta-regression of studies reporting associations of PD-L1 expression and clinical outcomes.

Study subgroup	Overall response rate			Progression-free survival			Overall survival		
	No. of studies	Pooled odds ratio (95% CI)	Meta-regression, P-value	No. of studies	Pooled hazard ratio (95% CI)	Meta-regression, P-value	No. of studies	Pooled hazard ratio (95% CI)	Meta-regression, P-value
Total	25	1.561 (0.941-2.589)		24	0.542 (0.414-0.710)		21	0.577 (0.465-0.716)	
Region			0.232			0.192			0.779
Eastern	21	1.514 (0.867-2.642)		21	0.505 (0.378-0.676)		19	0.583 (0.465-0.730)	
Western	2	6.001 (0.672-53.573)		3	0.863 (0.408-1.829)		2	0.523 (0.255-1.071)	
Target			**<0.001**			**0.003**			NA
PD-1	18	2.966 (1.835-4.793)		21	0.526 (0.413-0.671)		19	0.589 (0.474-0.733)	
PD-L1	7	0.424 (0.223-0.805)		1	3.340 (1.013-11.015)		0	NA	
Line of therapy			0.294			0.121			0.306
1^st^	8	1.094 (0.484-2.477)		7	0.386 (0.231-0.646)		7	0.476 (0.302-0.749)	
2^nd^ or later	16	1.944 (0.969-3.899)		14	0.628 (0.445-0.886)		12	0.626 (0.486-0.808)	
Intervention			0.904			0.056			**0.019**
Monotherapy	10	1.637 (0.678-3.952)		6	0.759 (0.480-1.199)		6	0.748 (0.550-1.019)	
Combination therapy	15	1.531 (0.806-2.909)		17	0.442 (0.323-0.605)		15	0.452 (0.335-0.610)	
PD-L1 scoring method			0.107			0.593			0.208
TPS	11	1.563 (0.863-2.832)		9	0.557 (0.356-0.871)		7	0.705 (0.491-1.014)	
CPS	10	3.168 (1.706-5.885)		13	0.483 (0.334-0.698)		13	0.508 (0.384-0.672)	
PD-L1 cutoff			0.723			**0.017**			**0.015**
1%	23	1.592 (0.932-2.718)		17	0.591 (0.442-0.791)		15	0.647 (0.508-0.823)	
5% or higher	1	2.429 (0.249-23.701)		5	0.309 (0.176-0.543)		5	0.304 (0.174-0.531)	

CI, confidence intervals, CPS, Combined positive score; NA, not available; TPS, Tumor proportion score.

The bold font signifies P-value<0.05.

### 3.7 Publication bias

Visual inspection of the funnel plots and clinical outcomes from included studies did not suggest asymmetry. Statistical analysis using Egger’s test showed similar results, confirming the absence of publication biases in these analyses (*P*=0.332, *P*=0.939, and *P*=0.133, respectively). Meanwhile, the funnel plot of OS revealed asymmetry, which was supported by a *P*-value of 0.002 on Egger’s test, indicating publication bias (**Supplementary Figure 3**).

## 4 Discussion

This meta-analysis revealed no significant differences in ORR and DCR between PD-L1 (+) and PD-L1 (-) patients with BTC undergoing anti-PD-1/PD-L1 therapy. However, PD-L1 (+) patients showed longer PFS and OS compared to that in PD-L1 (-) patients. These findings suggest the potential of PD-L1 expression as a helpful biomarker for predicting survival but not treatment responsiveness in patients with advanced BTC. Notably, clinical values of PD-L1 expression were significantly affected by the target of immunotherapy, presence of combined treatment, and PD-L1 cut-off values.

To the best of our knowledge, this study is the first meta-analysis evaluating the predictive value of PD-L1 expression in patients with BTC undergoing anti-PD-1/PD-L1 therapy. In immunotherapy-naïve patients with BTC, PD-L1 expression has been associated with tumor aggressiveness and poor prognosis (52–54). However, with the emergence of cancer immunotherapy, PD-L1 expression has been reported to be a potential predictive biomarker in various types of malignancies (16, 55, 56). Our findings similarly suggest that PD-L1 (+) expression was associated with prolonged PFS and OS, indicating its promise as a predictive biomarker in BTC. While the TOPAZ-1 trial, a representative phase III trial of immunotherapy for BTC, was excluded from this meta-analysis due to lacking data on direct comparisons between PD-L1 (+) and PD-L1 (-) patients within the experimental (durvalumab) group, the OS improvement of durvalumab compared to placebo has been reported to be more pronounced in the PD-L1(+) subset rather than the PD-L1(-) subset (HR, 0.79 *vs.* 0.86) (10). Considering that positive PD-L1 expression has been regarded as a poor prognostic factor in the absence of immunotherapy, our findings solidify the need for immunotherapy in PD-L1 (+) patients, allowing the prognostication of favorable clinical outcomes in this cohort. In contrast, PD-L1 expression did not show a significant association with ORR and DCR in this study. The reasons for the observed lack of correlation between PD-L1 expression and radiologic assessment, such as ORR and DCR, are unclear. Despite this, some underlying mechanisms have been proposed. Primarily, immunotherapies often induce robust inflammatory and immune responses within the tumor microenvironment. These dynamic reactions can result in intricate radiological patterns, posing challenges in the accurate assessment of tumor changes (e.g., morphology, size, metabolic activity). Additionally, BTC exhibits significant anatomical heterogeneity and inherent desmoplastic stroma, making it difficult to accurately delineate the tumor boundaries radiologically. This difficulty may result in the discrepancies between imaging assessments and survival prognosis.

Our findings indicate that PD-L1 may not be an absolute biomarker for immunotherapy response in patients with BTC. The difference in pooled ORR values for anti-PD-1/PD-L1 therapy between PD-L1 (+) and PD-L1 (-) patients was <5% (28.8% *vs.* 24.0%). Moreover, durable clinical benefits were reported even in PD-L1 (-) individuals. Therefore, PD-L1 expression in patients with BTC is more appropriately used as a biomarker to predict prognosis, rather than as an independent marker in determining the choice of anti-PD-1/PD-L1 therapy. Interestingly, the predictive value of PD-L1 expression significantly decreased in patients receiving anti-PD-L1 agents compared to those receiving anti-PD-1 agents. This trend has also been observed in carcinomas other than BTC (17, 56, 57). Further studies are warranted to verify whether the predictive value of PD-L1 expression differs depending on the target of ICIs (PD-1 *vs.* PD-L1) in cancer immunotherapy.

Various drugs, including conventional chemotherapeutic agents, anti-angiogenic agents, and inhibitors targeting other immune checkpoints (e.g., cytotoxic T lymphocyte-4), have been introduced in combination regimens with anti-PD-1/PD-L1 agents for the treatment of BTC (27, 28, 30, 37). A recent meta-analysis involving patients with BTC showed superior treatment response and clinical outcomes using combination regimens with anti-PD-1/PD-L1 therapy compared to anti-PD-1/PD-L1 monotherapy (58). This was consistent with our results, which demonstrated a higher response rate in combination therapy. Our study also showed that the role of PD-L1 expression as a predictive biomarker in OS was more prominent in anti-PD-1/PD-L1-based combination therapy than in monotherapy. Promising results of recent phase 3 studies strongly indicate that ICI-based combination therapy will likely become the first-line standard treatment for advanced BTC (10, 11). As such, future studies should further explore PD-L1 expression as a biomarker in combination therapy.

Although various methods in IHC have been reported for evaluating PD-L1 expression, the optimal method in patients with BTC remain unclear. Different PD-L1 antibodies, staining platforms, scoring methods, and cut-offs have been used in clinical trials and practices. Dako’s 22C3 and Ventana’s SP263 are two major antibodies commonly used in PD-L1 IHC, with more than half of the included studies reporting the use of these assays. As reported in other cancer types, both assays showed high concordance and may be utilized interchangeably (59). For PD-L1 scoring methods, TPS and CPS were the most frequently used for BTC studies. According to subgroup analyses and meta-regression in our study, scoring method did not significantly affect the predictive or prognostic value of PD-L1 expression in BTC. Conversely, when PD-L1 expression was determined at a high cut-off level (≥5%), the predictive value of survival was greatly improved. In a recent study including only PD-L1 (+) patients with BTC, higher PD-L1 values (≥50%) were associated with better therapeutic response to pembrolizumab compared to the lower PD-L1 values (1% to <50%) (60). Thus, similar to the findings in non-small cell lung cancer, further research is required to assess the prognosis of patients with BTC using subgroup analyses based on PD-L1 expression levels (61).

Given the limitations of PD-L1 expression as the only biomarker for immunotherapy in BTC, efforts have been made to identify other promising biomarkers. The overall number of somatic mutations in a tumor cell, referred to as “tumor mutation burden (TMB),” is an alternative candidate, since tumors with high TMB are expected to be more immunogenic. According to a study by Chiang et al., higher TMB (top 20%; ≥7.1 mut/Mb) predicted prolonged PFS but not OS in patients receiving chemotherapy plus nivolumab (34). However, data regarding TMB in BTC are limited, and previous studies have employed arbitrary and different cut-off thresholds (27, 36, 38). Another potential predictive biomarker for ICI responsiveness is microsatellite instability (MSI). Despite its modest predictive value for ICI response in most solid malignancies, MSI-high BTC seems to be quite rare, with a prevalence of 1-2% (34, 60, 62). Recently, a retrospective study reported that hematologic parameters, such as neutrophil-to-lymphocyte ratio or inflammatory cytokines, can be used for predicting therapeutic response to anti-PD-1 therapy in BTC (63). Since existing biomarkers, including PD-L1, have not yet shown satisfactory results, the identification of other validated predictors of immunotherapy response is equally important.

Despite the insights offered by this study, certain limitations should be acknowledged. First, significant heterogeneity was noted in pooled analyses, which was likely due to variations in treatment modalities and the lack of standardized analytical methods for PD-L1 IHC. Subgroup analyses and meta-regression were undertaken to address this matter; however, the persisting heterogeneity may still influence the results. Prospective biomarker studies conducted with uniform criteria for PD-L1 IHC are recommended to address this issue. Second, relevant studies might have been excluded due to lacking data on biomarker analysis or missing reports due to non-significant results. Third, the inclusion of several retrospective studies may have introduced bias. To address this, sensitivity analysis of prospective studies was performed for the primary outcomes. Lastly, while BTC exhibits diverse prognoses depending on its location, this meta-analysis was unable to provide the predictive value of PD-L1 according to location due to insufficient information from individual studies. Ultimately, a prospective combination of deep sequencing and experimental study is indispensable for biomarker research in immunotherapy for BTC.

In conclusion, PD-L1 expression may be a helpful predictive biomarker for survival in patients with BTC undergoing anti-PD-1/PD-L1 therapy. However, its utility as a biomarker for radiologic tumor response is less reliable. Various regimens of ICIs and the lack of standardized analytic methods can significantly affect the predictive value of PD-L1 expression. Therefore, we expect that further biomarker studies, including PD-L1, must be reported using highly efficacious regimens and established IHC methods to fully support the use of anti-PD-1/PD-L1 therapy in BTC.

## Data availability statement

The original contributions presented in the study are included in the article/**Supplementary Material**. Further inquiries can be directed to the corresponding author.

## Author contributions

SY: Writing – original draft, Validation, Software, Methodology, Investigation, Formal analysis, Conceptualization. SW: Writing – original draft, Validation, Supervision, Formal analysis, Conceptualization. JC: Writing – original draft, Formal analysis, Data curation. DK: Writing – review & editing, Investigation, Data curation. JK: Writing – review & editing, Supervision, Investigation. JP: Writing – review & editing, Investigation, Data curation. HS: Writing – review & editing, Methodology, Data curation. MC: Writing – review & editing, Supervision, Methodology. IC: Writing – review & editing, Investigation, Data curation. JH: Writing – review & editing, Methodology, Formal analysis.

## Supplementary material

Supplementary Figure 1 Forest plots showing the primary outcomes in total patients with biliary tract cancer treated with anti PD-1/PD-L1 therapy. **(A)** Objective response rate. **(B)** Disease control rate. **(C)** Grade 3-5 adverse event rate.

Supplementary Figure 2 Sensitivity analysis excluding retrospective studies showing the results of primary outcomes in patients with biliary tract cancer treated with anti PD-1/PD-L1 therapy according to PD-L1 expression. **(A)** Objective response rate. **(B)** Disease control rate. **(C)** Progression-free survival. **(D)** Overall survival.

Supplementary Figure 3 Funnel plot evaluation for publication bias. **(A)** Objective response rate. **(B)** Disease control rate. **(C)** Progression-free survival. **(D)** Overall survival.
