# Supplementary figures and images for: The predictive value of PD-L1 expression in response to anti-PD-1/PD-L1 therapy for biliary tract cancer: a systematic review and meta-analysis
Source: Front Immunol. 2024 Mar 28;15:1321813. doi: 10.3389/fimmu.2024.1321813 (PMC11007040; doi:10.3389/fimmu.2024.1321813)

## Slide 1
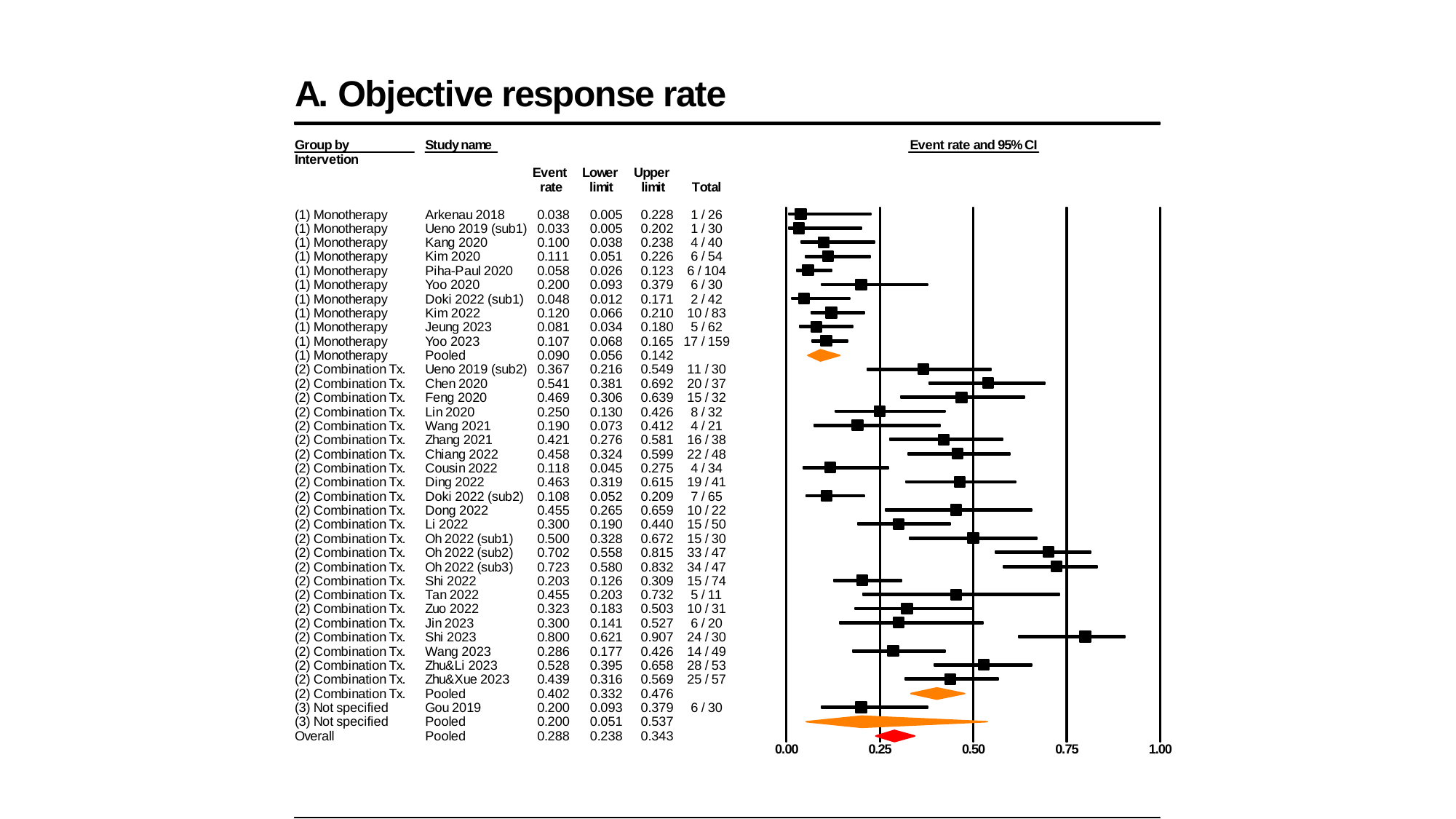

## Slide 2
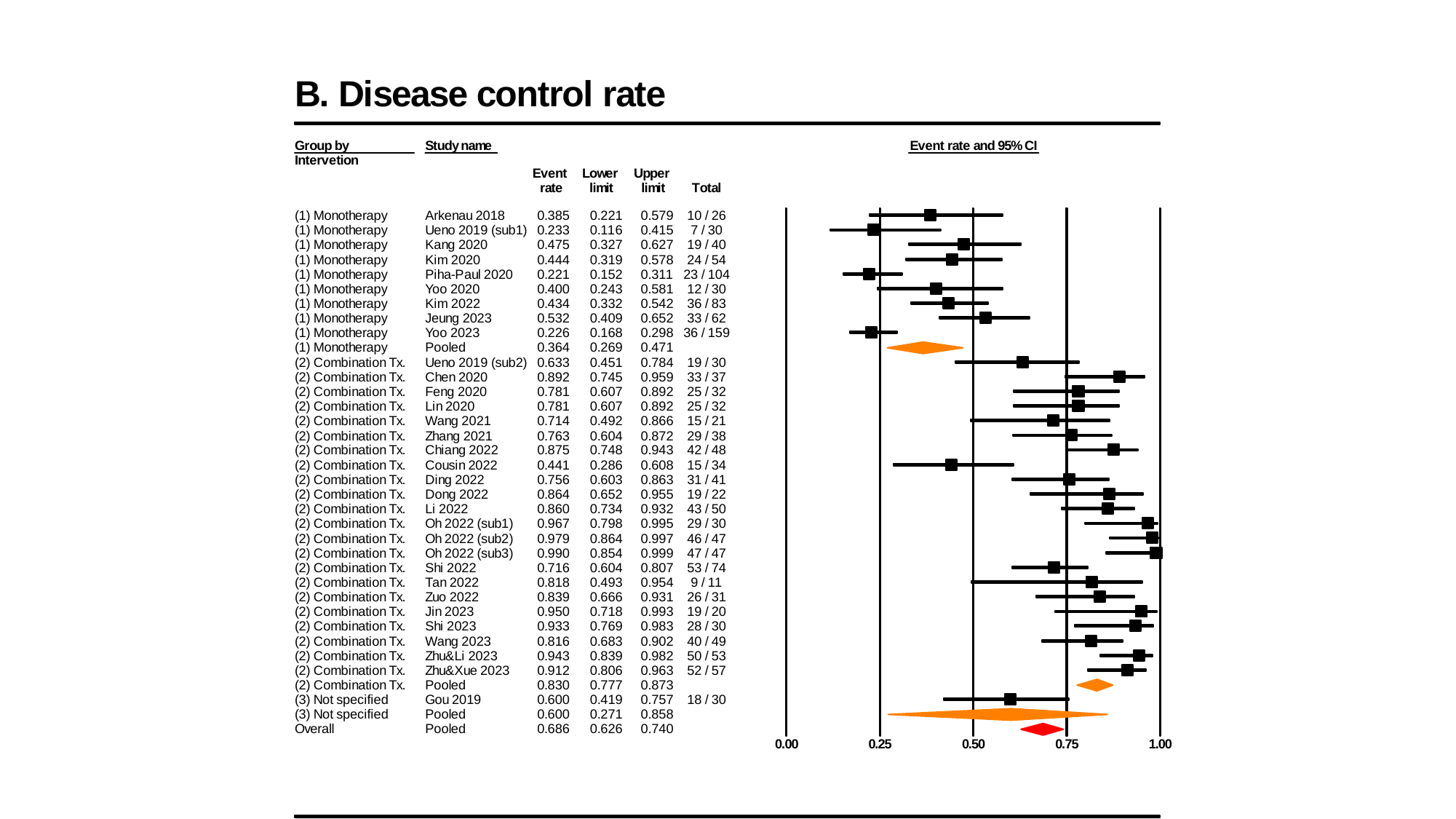

## Slide 3
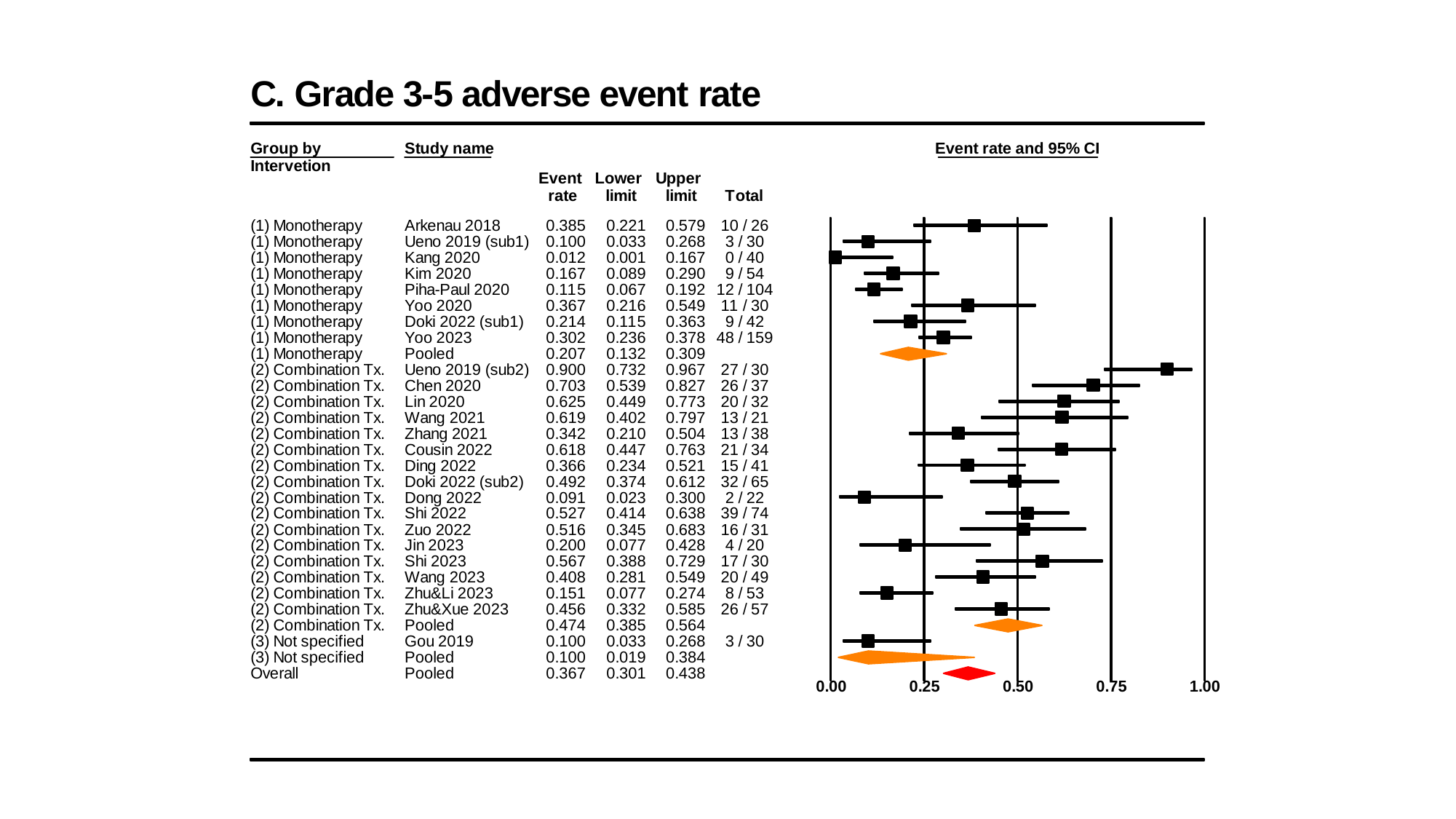

Supplement: Supplementary Figure 1 — Forest plots showing the primary outcomes in total patients with biliary tract cancer treated with anti PD-1/PD-L1 therapy. (A) Objective response rate. (B) Disease control rate. (C) Grade 3-5 adverse event rate. [file Presentation_1.pptx]

## Slide 1
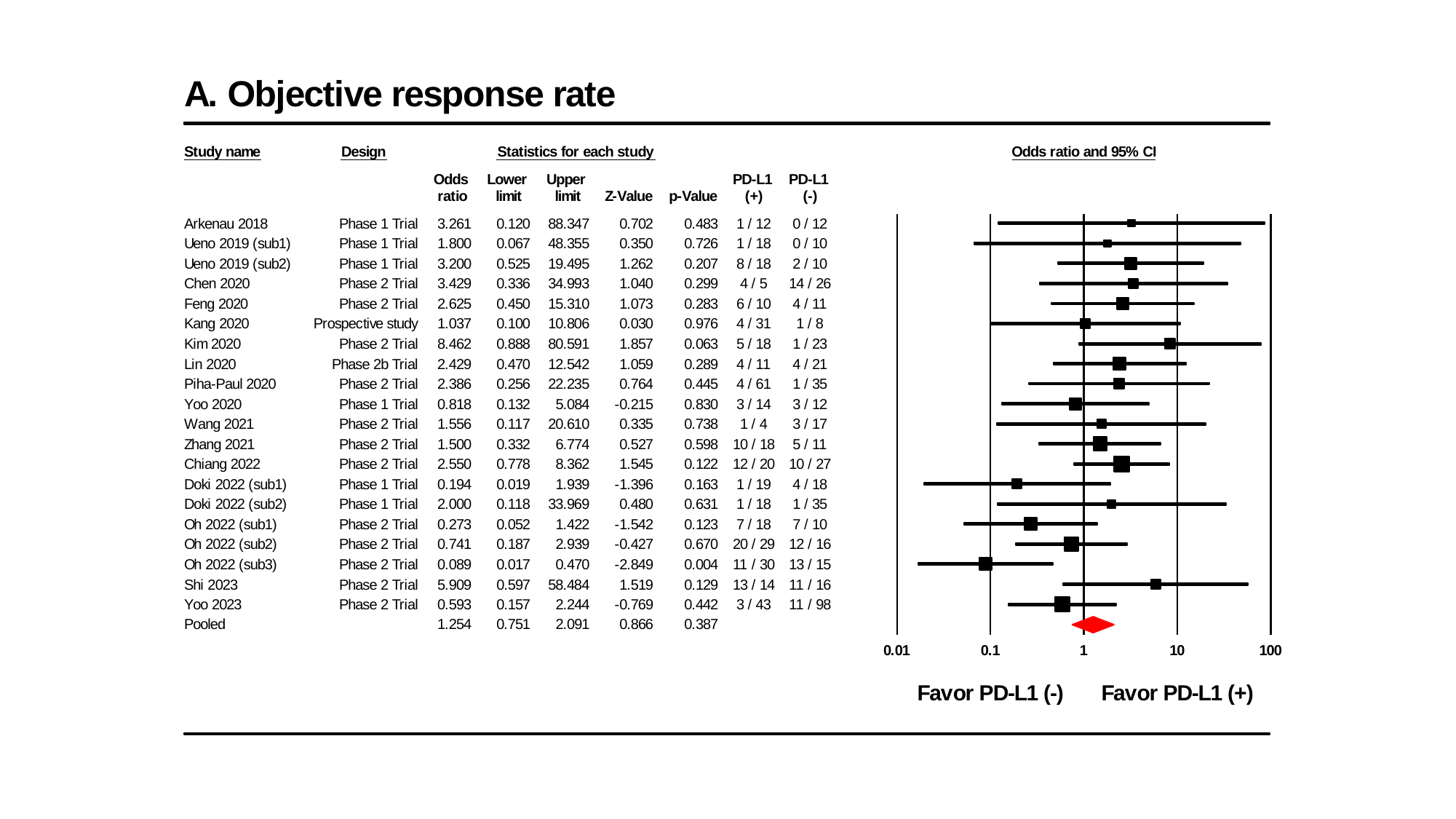

## Slide 2
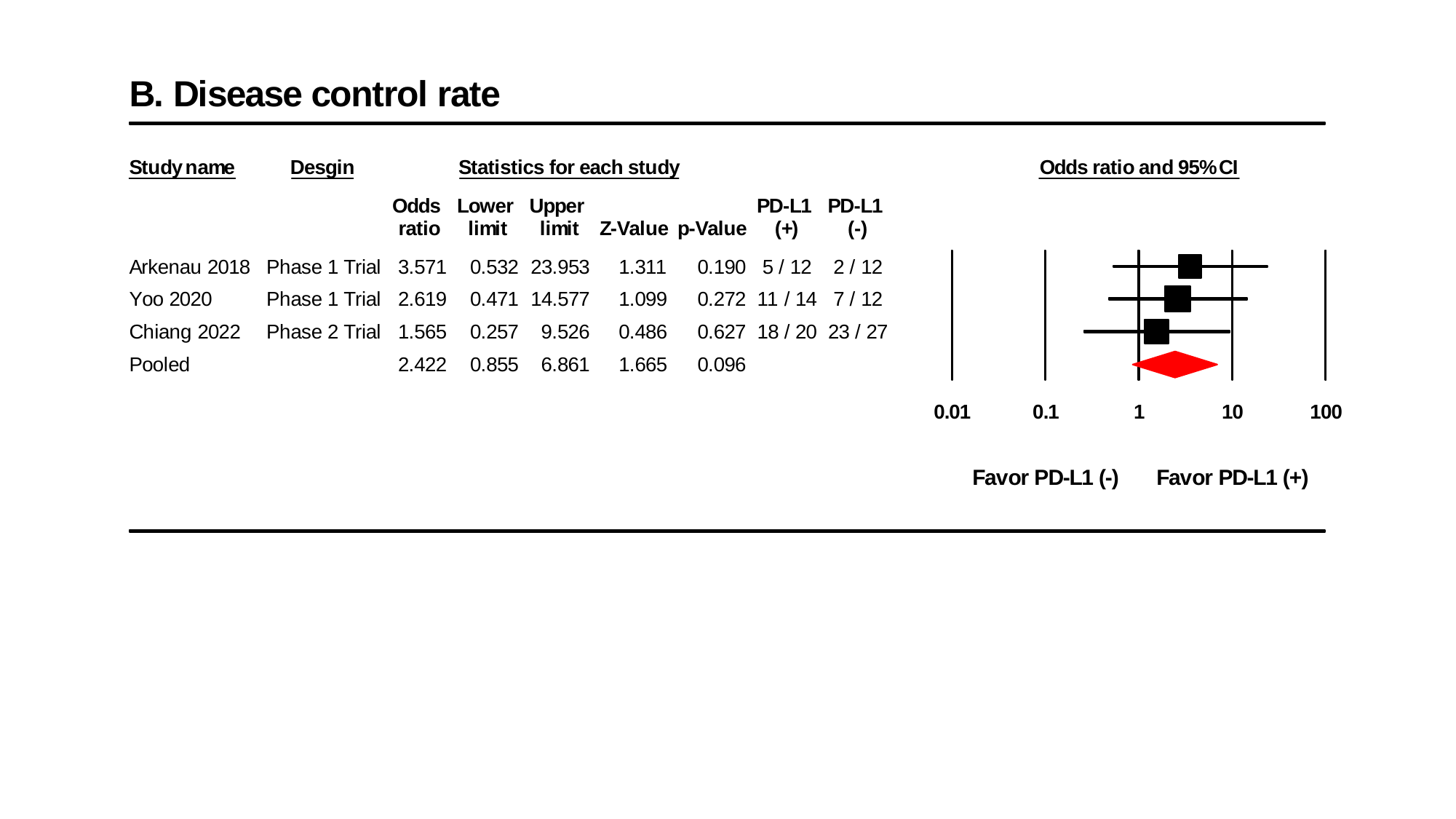

## Slide 3
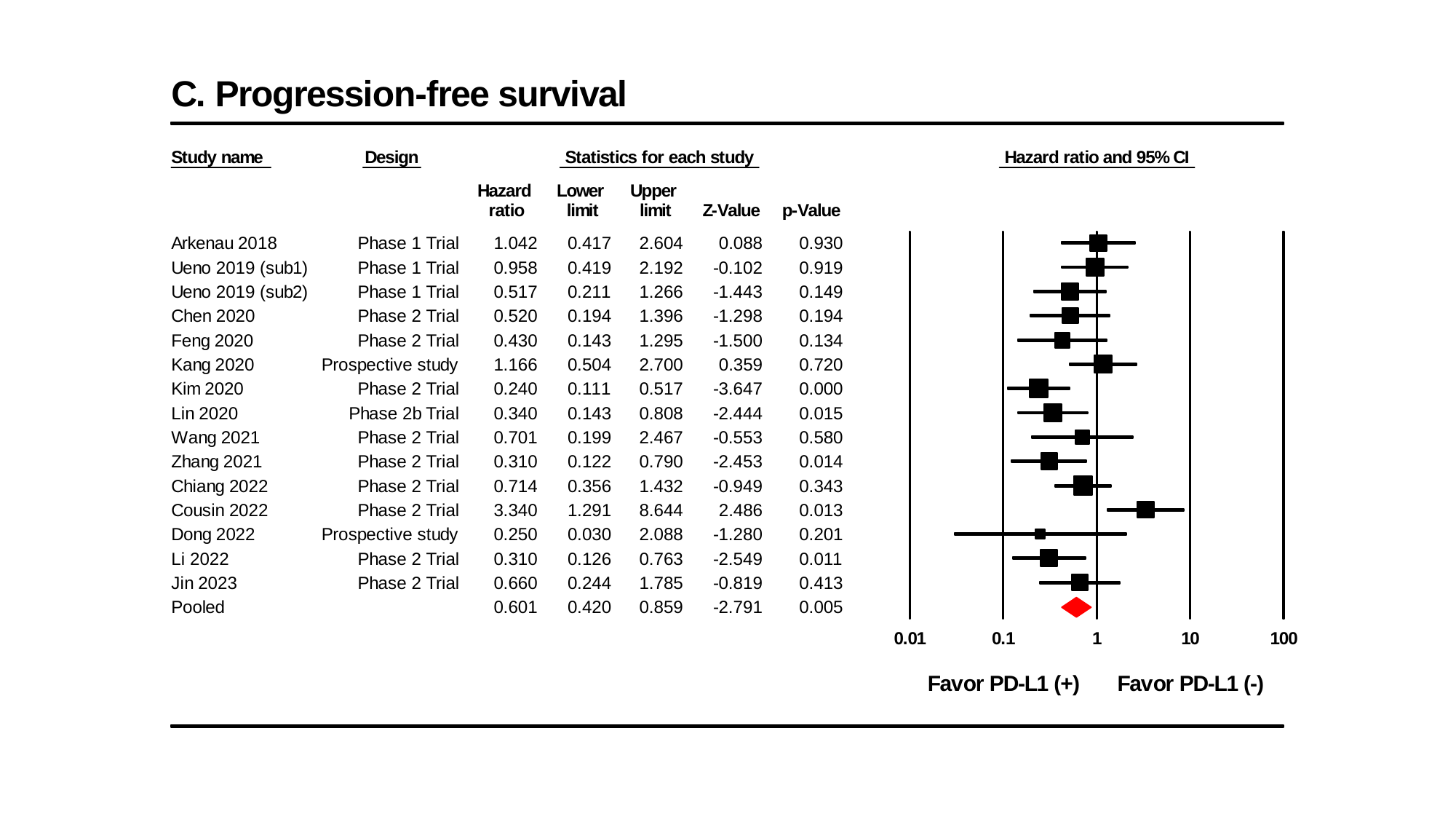

## Slide 4
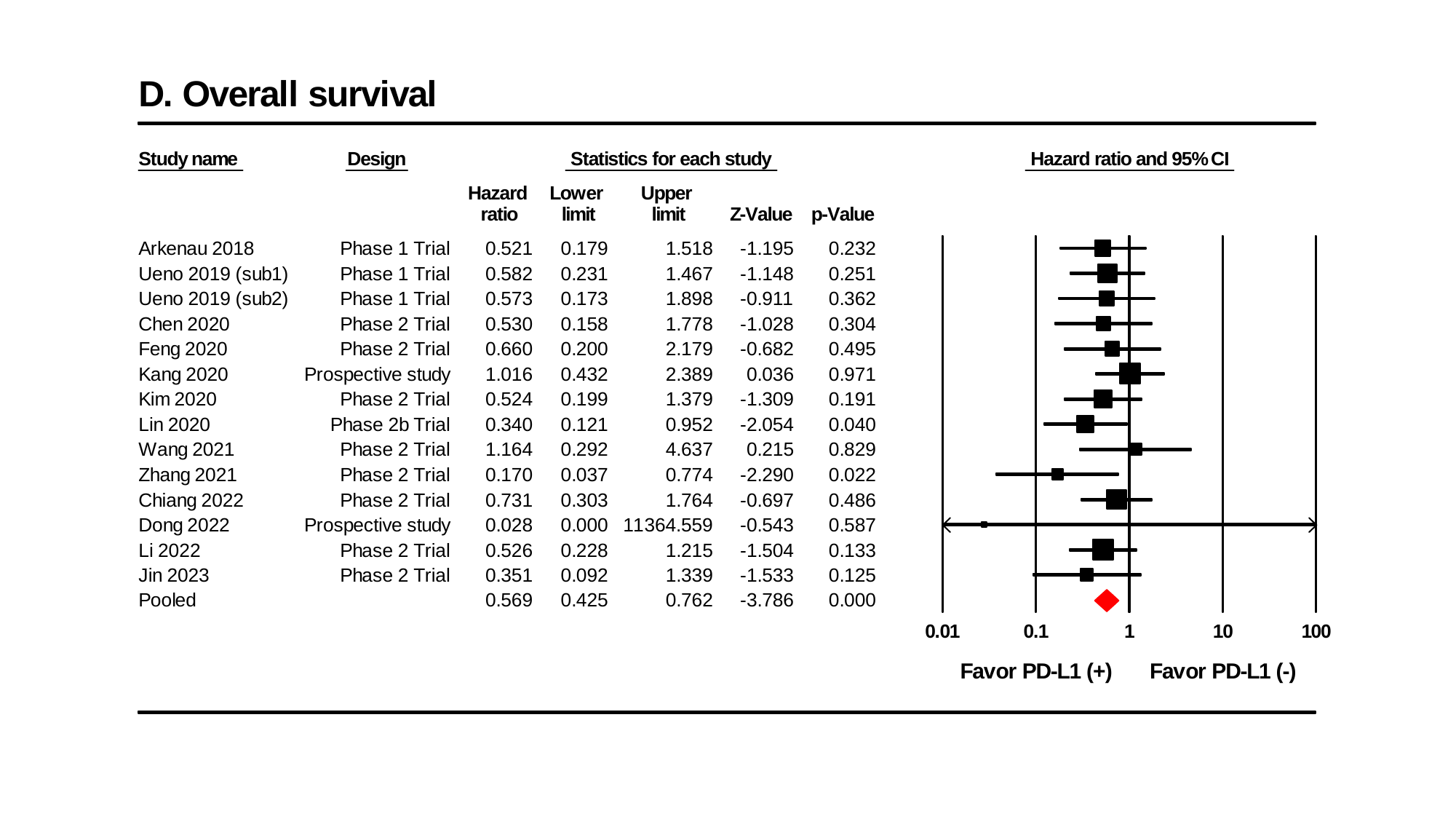

Supplement: Supplementary Figure 2 — Sensitivity analysis excluding retrospective studies showing the results of primary outcomes in patients with biliary tract cancer treated with anti PD-1/PD-L1 therapy according to PD-L1 expression. (A) Objective response rate. (B) Disease control rate. (C) Progression-free survival. (D) Overall survival. [file Presentation_2.pptx]
