# Supplementary material for: The predictive value of PD-L1 expression in response to anti-PD-1/PD-L1 therapy for biliary tract cancer: a systematic review and meta-analysis
Source: Front Immunol. 2024 Mar 28;15:1321813. doi: 10.3389/fimmu.2024.1321813 (PMC11007040; doi:10.3389/fimmu.2024.1321813)
Supplement: Supplementary Figure 3 — Funnel plot evaluation for publication bias. (A) Objective response rate. (B) Disease control rate. (C) Progression-free survival. (D) Overall survival. [file Presentation_3.pptx]

## Slide 1
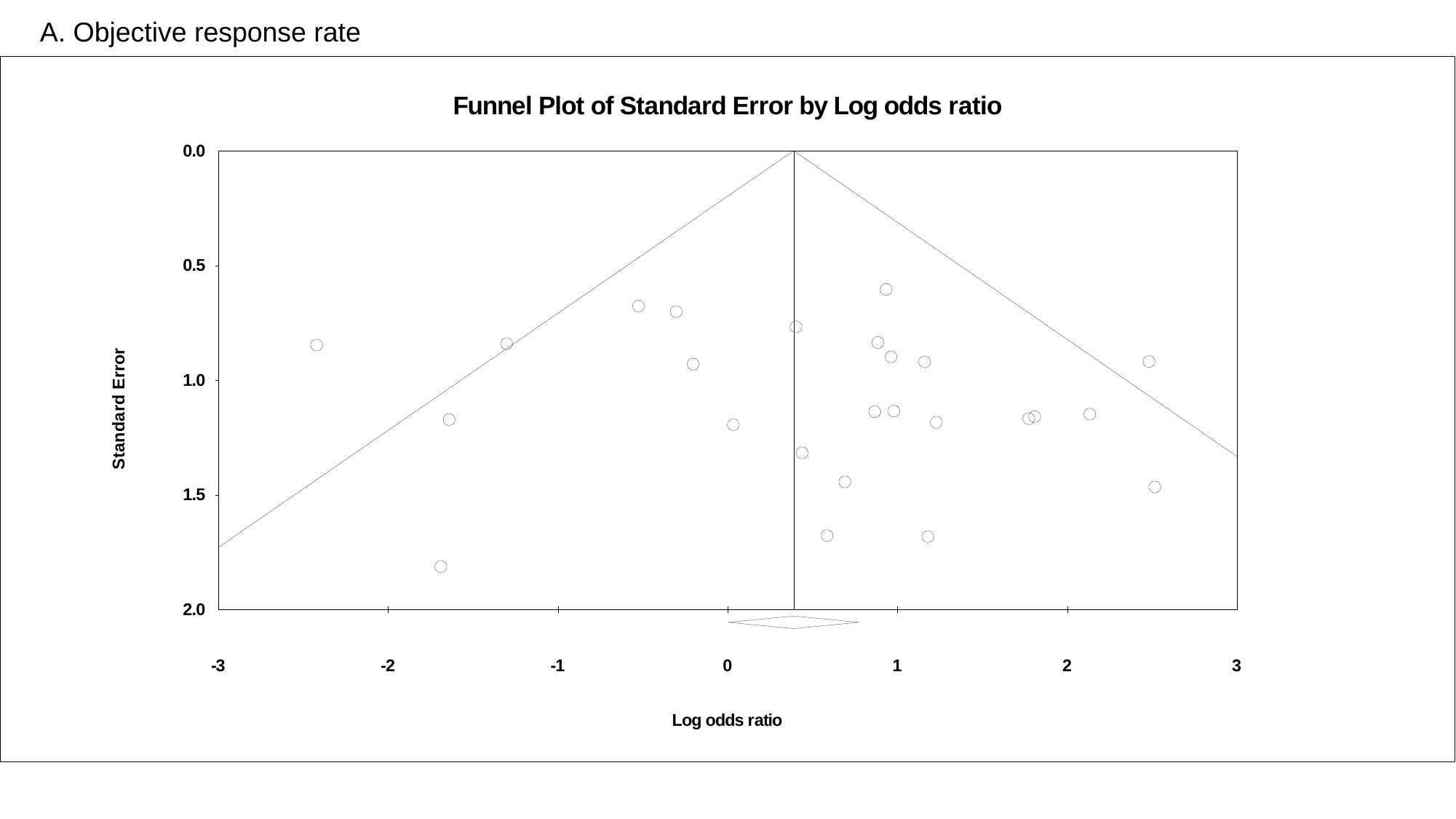

A. Objective response rate

## Slide 2
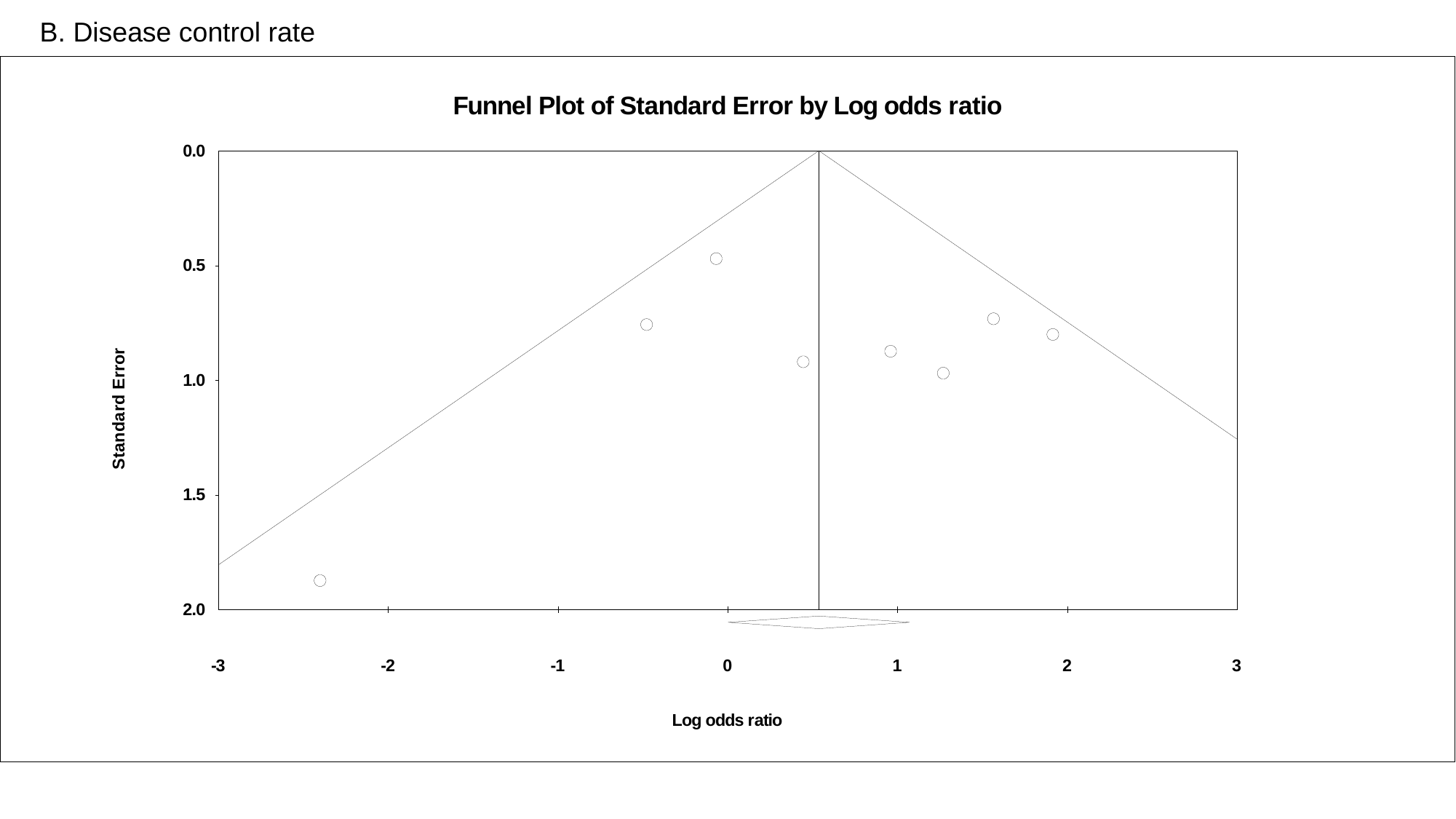

B. Disease control rate

## Slide 3
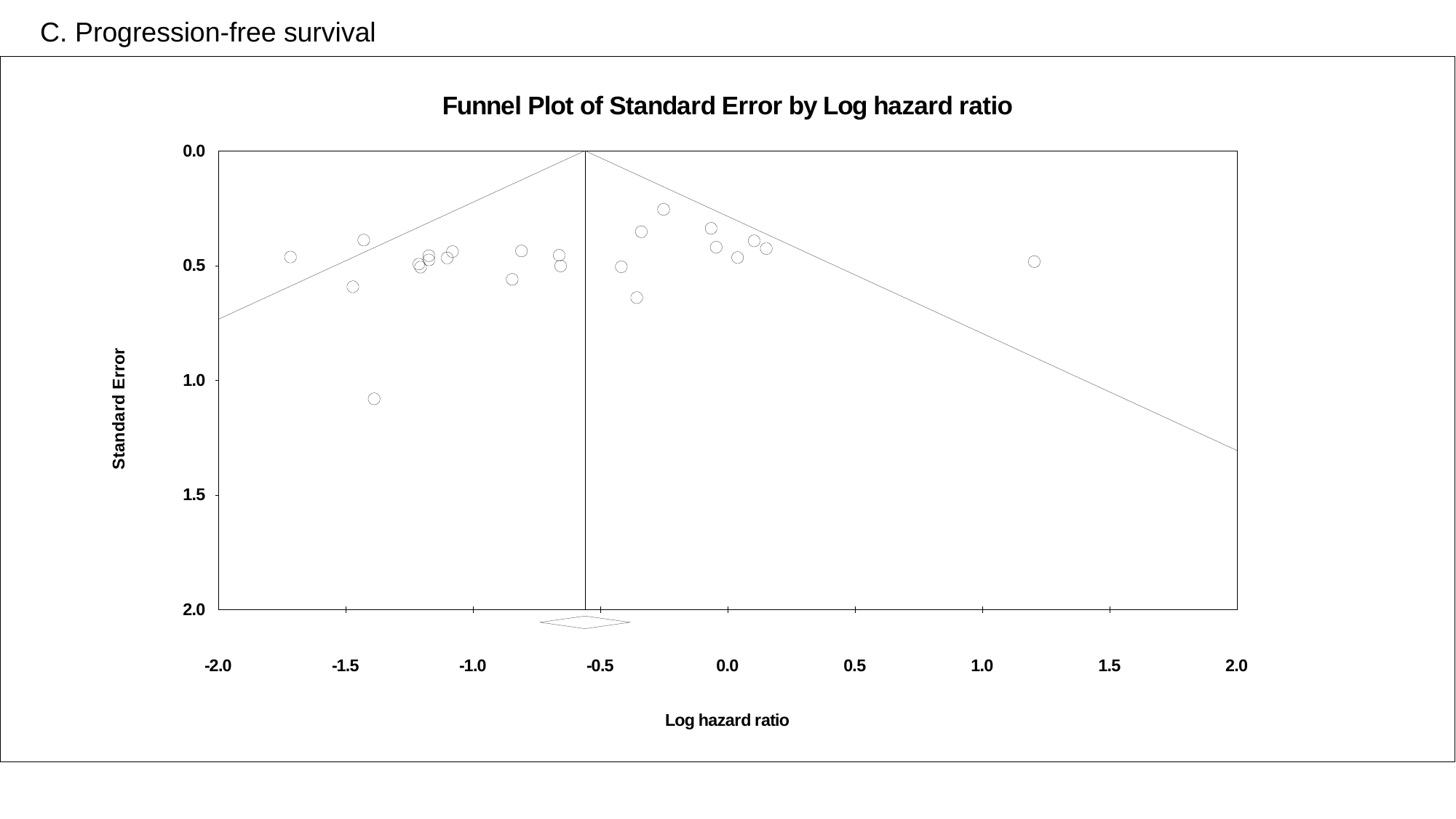

C. Progression-free survival

## Slide 4
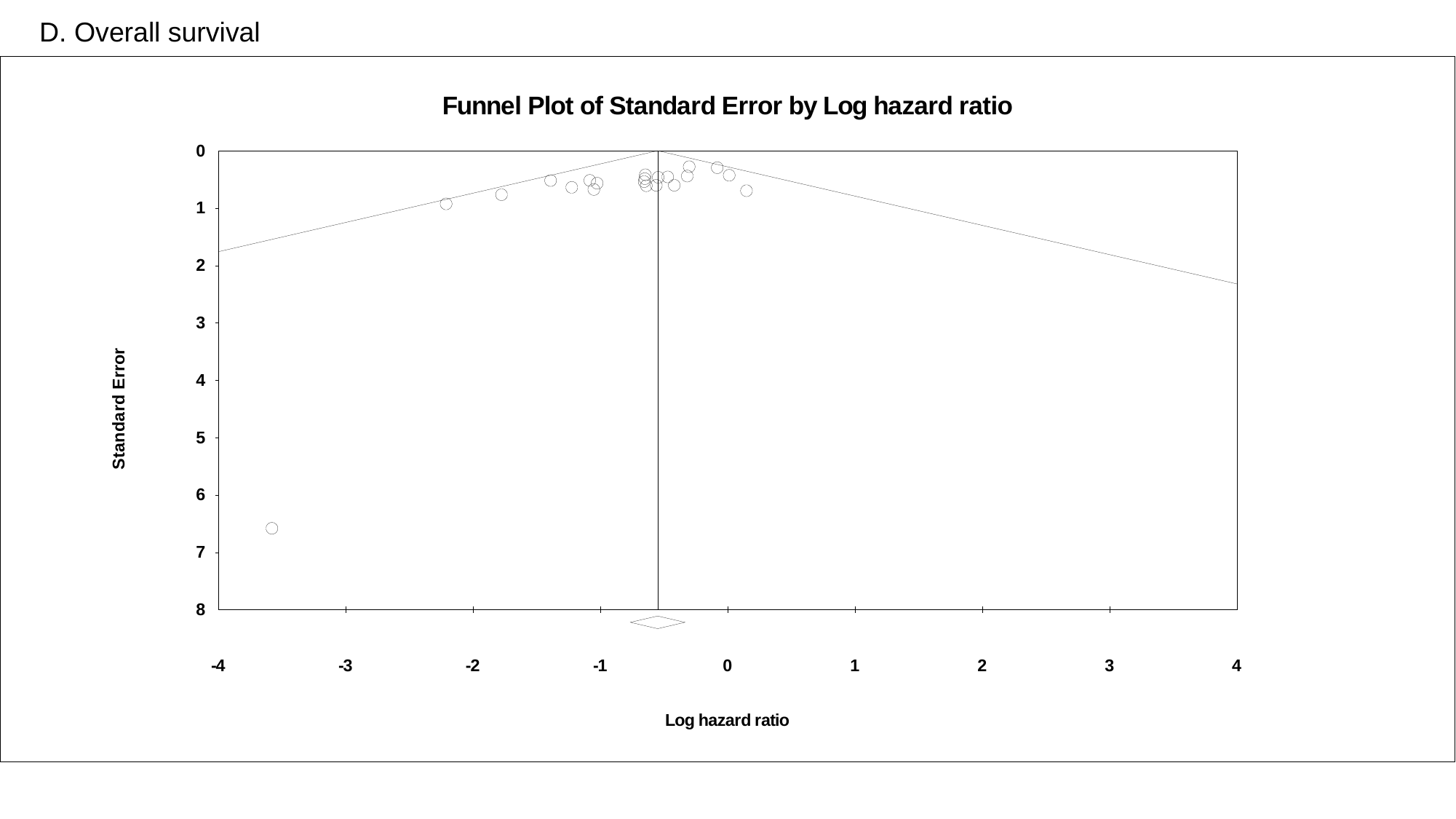

D. Overall survival
