# Supplementary material for: The predictive value of PD-L1 expression in response to anti-PD-1/PD-L1 therapy for biliary tract cancer: a systematic review and meta-analysis
Source: Front Immunol. 2024 Mar 28;15:1321813. doi: 10.3389/fimmu.2024.1321813 (PMC11007040; doi:10.3389/fimmu.2024.1321813)
Supplement: Supplementary file 5 [file Table_2.docx]

**Supplementary Table 2.** Research strategy.

**Pubmed**

| No. | Search detail |
| --- | --- |
| #1 | cholangiocarcinoma[MeSH Terms] OR bile duct neoplasms[MeSH Terms] OR gallbladder neoplasms[MeSH Terms] |
| #2 | cholangio*[Title/Abstract] OR “bile duct”[Title/Abstract] OR biliary[Title/Abstract] OR gallbladder[Title/Abstract] |
| #3 | carcinoma*[Title/Abstract] OR cancer*[Title/Abstract] OR tumor*[Title/Abstract] OR neoplasm*[Title/Abstract] OR malign*[Title/Abstract] |
| #4 | #2 AND #3 |
| #5 | #1 OR #4 |
| #6 | Immune Checkpoint Inhibitors[MeSH Terms] |
| #7 | “Immunocheckpoint inhibitor*”[Title/Abstract] OR “Immune checkpoint inhibitor*”[Title/Abstract] OR PD1[Title/Abstract] OR PDL1[Title/Abstract] OR PD-1[Title/Abstract] OR PD-L1[Title/Abstract] |
| #8 | #6 OR #7 |
| #9 | #5 AND #8 |
| Filters: English | |
| Results: 549 articles were found | |

**Embase**

| No. | Search detail |
| --- | --- |
| #1 | ‘biliary tract tumor’/exp |
| #2 | 'cholangio*':ti,ab,kw OR ('bile’ AND ‘duct’):ti,ab,kw OR 'biliary':ti,ab,kw OR 'gallbaldder':ti,ab,kw |
| #3 | ‘carcinoma*':ti,ab,kw OR ‘tumor*':ti,ab,kw OR ‘neoplasm*’:ti,ab,kw OR ‘cancer*’:ti,ab,kw OR ‘malign*’:ti,ab,kw |
| #4 | #2 AND #3 |
| #5 | #1 OR #4 |
| #6 | 'immune checkpoint inhibitor'/exp |
| #7 | ‘immunocheckpoint inhibitor*’:ti,ab,kw OR ‘immune checkpoint inhibitor*’:ti,ab,kw OR PD1:ti,ab,kw OR PDL1:ti,ab,kw OR ‘PD-1’:ti,ab,kw OR ‘PD-L1’:ti,ab,kw |
| #8 | #6 OR #7 |
| #9 | #5 AND #8 |
| #10 | #9 AND [english]/lim |
| Results: 1561 articles were found | |

**The Cochrane library**

| No. | Search detail |
| --- | --- |
| #1 | Mesh descriptor: [Cholangiocarcinoma] explored all trees |
| #2 | Mesh descriptor: [Bile duct Neoplasms] explored all trees |
| #3 | Mesh descriptor: [Gallbladder Neoplasms] explored all trees |
| #4 | 'cholangio*':ti,ab,kw OR ('bile’ AND ‘duct’):ti,ab,kw OR 'biliary':ti,ab,kw OR 'gallbaldder':ti,ab,kw |
| #5 | ‘carcinoma*':ti,ab,kw OR ‘tumor*':ti,ab,kw OR ‘neoplasm*’:ti,ab,kw OR ‘cancer*’:ti,ab,kw OR ‘malign*’:ti,ab,kw |
| #6 | #4 and #5 |
| #7 | #1 OR #2 OR #3 OR #6 |
| #8 | MeSH descriptor: [Immune Checkpoint Inhibitors] explode all trees |
| #9 | ‘immunocheckpoint inhibitor*’:ti,ab,kw OR ‘immune checkpoint inhibitor*’:ti,ab,kw OR PD1:ti,ab,kw OR PDL1:ti,ab,kw OR ‘PD-1’:ti,ab,kw OR ‘PD-L1’:ti,ab,kw |
| #10 | #8 OR #9 |
| #11 | #7 AND #10 |
| Results: 77 articles were found | |
