# Supplementary material for: The predictive value of PD-L1 expression in response to anti-PD-1/PD-L1 therapy for biliary tract cancer: a systematic review and meta-analysis
Source: Front Immunol. 2024 Mar 28;15:1321813. doi: 10.3389/fimmu.2024.1321813 (PMC11007040; doi:10.3389/fimmu.2024.1321813)
Supplement: Supplementary file 6 [file Table_3.docx]

**Supplementary Table 3**. Risk of bias assessment of included studies for prognostic analysis based on the Quality In Prognosis Studies tool.

| Study | Study participation | Study Attrition | Prognostic Factor | Outcome Measurement | Study Confounding | Statistical Analysis and Reporting |
| --- | --- | --- | --- | --- | --- | --- |
| Arkenau 2018 | L | L | L | L | L | M |
| Gou 2019 | H | M | M | H | H | M |
| Ueno 2019 | L | L | L | L | L | M |
| Chen 2020 | L | L | M | L | L | L |
| Feng 2020 | L | L | L | L | L | L |
| Kang 2020 | M | L | L | L | L | M |
| Kim 2020 | L | L | M | L | L | M |
| Lin 2020 | L | L | L | L | L | L |
| Piha-Paul 2020 | L | L | L | H | L | L |
| Yoo 2020 | L | L | L | M | L | L |
| Wang 2021 | L | L | L | H | L | M |
| Zhang 2021 | L | L | L | M | H | L |
| Chiang 2022 | L | L | H | M | M | M |
| Cousin 2022 | L | L | L | H | L | L |
| Ding 2022 | H | L | L | L | L | L |
| Doki 2022 | L | M | L | L | L | L |
| Dong 2022 | M | L | H | M | H | M |
| Kim 2022 | H | L | L | L | M | M |
| Li 2022 | L | L | L | L | L | M |
| Oh 2022 | L | L | M | H | L | L |
| Shi 2022 | H | L | L | L | H | L |
| Tan 2022 | H | M | M | M | H | L |
| Zuo 2022 | H | L | H | L | H | L |
| Jeong 2023 | H | M | L | L | M | M |
| Jin 2023 | L | L | L | L | L | M |
| Shi 2023 | L | L | L | H | L | L |
| Wang 2023 | H | L | L | L | H | L |
| Yoo 2023 | L | L | L | H | L | L |
| Zhu&Li 2023 | H | L | M | M | M | L |
| Zhu&Xue 2023 | H | L | M | L | H | M |

H: High risk of bias; M: Moderate risk of bias; L: Low risk of bias.
