# Supplementary material for: The predictive value of PD-L1 expression in response to anti-PD-1/PD-L1 therapy for biliary tract cancer: a systematic review and meta-analysis
Source: Front Immunol. 2024 Mar 28;15:1321813. doi: 10.3389/fimmu.2024.1321813 (PMC11007040; doi:10.3389/fimmu.2024.1321813)
Supplement: Supplementary file 7 [file Table_4.docx]

**Supplementary Table 4**. Main outcomes of the total cohort extracted from included studies.

| Study | Sample size, n | Median age, years (range) | Male, % | Median follow-up, months | CR, % | PR, % | SD, % | PD, % | mPFS (95% CI), months | mOS (95% CI), months | Grade 3–4 AEs, % |
| --- | --- | --- | --- | --- | --- | --- | --- | --- | --- | --- | --- |
| Arkenau 2018 | 26 | 63 (36-78) | 69.2 | 15.7 | 0.0 | 3.8 | 34.6 | 50.0 | 1.6 (1.4-2.7) | 6.4 (4.2-13.3) | 38 |
| Gou 2019 | 30 | 53 (36-80) | 60.0 | NR | 3.3 | 16.7 | 40.0 | 40.0 | 3.1 (2.1-4.1) | NR | 10 |
| Ueno 2019 (sub1) | 30 | 68 (60–71) | 60.0 | 5.1 | 0.0 | 3.3 | 20.0 | 73.3 | 1.4 (90% CI, 1.4-1.4) | 5.2 (90% CI, 4.5-8.7) | 10 |
| Ueno 2019 (sub2) | 30 | 68 (54–75) | 67.5 | 8.2 | 0.0 | 36.7 | 26.7 | 30.0 | 4.2 (90% CI, 2.8-5.6) | 15.4 (90% CI, 11.8-NE) | 90 |
| Chen 2020 | 37 | 64 (41–74) | 70.3 | 11.8 | 0.0 | 54.1 | 35.1 | NR | 6.1 (5.1-6.8) | 11.8 (8.3-15.4) | 70 |
| Feng 2020 | 32 | 60 (27-69) | 56.0 | 12.8 | 15.6 | 31.3 | 31.3 | 6.3 | 6.1 (3.4-7.9) | 8.5 (5.0-12.5) | NR |
| Kang 2020 | 40 | 66 (43-83) | 56.9 | 8.2 | 0.0 | 10.0 | 37.5 | 50.0 | 1.5 (0.0-3.0) | 4.3 (3.5-5.1) | 0 |
| Kim 2020 | 54 | 65 (28-86) | 50.0 | 12.4 | 0.0 | 11.1 | 33.3 | 42.6 | 3.7 (2.3-5.7) | 14.2 (6.0-NE) | 17 |
| Lin 2020 | 32 | 57 (IQR, 50–69) | 56.0 | 9.5 | 0.0 | 25.0 | 53.1 | 21.9 | 4.9 (4.7-5.2) | 11 (9.6-12.3) | 62 |
| Piha-Paul 2020 | 104 | 61 (41-76) | 57.5 | 7.5 | 0.0 | 5.8 | 16.3 | 62.5 | 2.0 (1.9-2.1) | 7.4 (5.5-9.6) | 12 |
| Yoo 2020 | 30 | 67 (IQR, 58-69) | 63.0 | 15.3 | 6.7 | 13.3 | 20.0 | 53.3 | 2.5 (1.3-5.6) | 12.7 (6.7-15.7) | 37 |
| Wang 2021 | 21 | 60 (39-72) | 52.4 | 13.4 | 0.0 | 19.0 | 52.4 | 28.6 | 4.4 (2.4-6.3) | 13.1 (8.1-18.2) | 63 |
| Zhang 2021 | 38 | 63 (57-65) | 37.0 | 13.7 | 0.0 | 42.1 | 34.2 | 23.7 | 8.0 (4.6-11.4) | 17.7 (NE) | 34 |
| Chiang 2022 | 48 | 66 (30-80) | 46.0 | 6.4 | 2.1 | 43.8 | 41.7 | 10.4 | 8.0(5.8-NE) | Not reached | NR |
| Cousin 2022 | 34 | 63 (36-80) | 58.0 | 9.8 | 0.0 | 11.8 | 32.4 | 41.2 | 2.5 (1.9-5.5) | 11.9 (6.2-NE) | 62 |
| Ding 2022 | 41 | 59 (33-75) | 61.0 | 12.1 | 0.0 | 46.3 | 29.3 | 24.4 | 6.6 (4.9-8.3) | 16.6 (5.0-28.2) | 37 |
| Doki 2022 (sub1) | 42 | 64 (NR) | NR | NR | 0.0 | 4.8 | NR | NR | 1.5 (1.4-2.6) | 8.1 (5.6-10.1) | 21 |
| Doki 2022 (sub2) | 65 | 62 (NR) | NR | NR | 0.0 | 10.8 | NR | NR | 1.6 (1.4-2.8) | 10.1 (6.5-11.6) | 49 |
| Dong 2022 | 22 | 58 (40-81) | 54.5 | 13.2 | 0.0 | 45.5 | 40.9 | 13.6 | 9.9 (1.2-17.1) | 11.8 (4.6-17.1) | 9 |
| Kim 2022 | 83 | 64 (35-84) | 65.1 | 4.8 | 1.2 | 10.8 | 31.3 | 45.8 | NR | NR | NR |
| Li 2022 | 50 | 62 (32–75) | 56.0 | 24 | 2.0 | 28.0 | 56.0 | 12.0 | 7.0 (5.0-8.9) | 15.0 (11.6-18.4) | NR |
| Oh 2022 (sub1) | 30 | 64 (IQR, 57-68) | 57.0 | 28.5 | 6.7 | 43.3 | 46.7 | 3.3 | 12.8 (10.1-15.4) | 15.0 (10.7-19.2) | NR |
| Oh 2022 (sub2) | 47 | 66 (IQR, 60-71) | 53.0 | 11.9 | 2.1 | 68.1 | 27.7 | 2.1 | 12.3 (9.3-15.2) | 18.7 (14.1-23.2) | NR |
| Oh 2022 (sub3) | 47 | 61 (IQR, 57-71) | 40.0 | 11.3 | 6.4 | 66.0 | 27.7 | 0.0 | 11.8 (6.9-16.6) | 20.2 (12.8-27.6) | NR |
| Shi 2022 | 74 | 63 (43–78) | 60.8 | 15 | 0.0 | 20.3 | 51.4 | 17.6 | 4.0 (3.5-5.0) | 9.5 (9.0-11.0) | 52 |
| Tan 2022 | 11 | 57 (53-67) | 36.4 | NR | 9.1 | 36.4 | 36.4 | 18.2 | 7.5 (2.5-12.5) | 12.7 (5.5-19.5) | NR |
| Zuo 2022 | 31 | 62 (IQR, 58-69) | 45.2 | 8 | 0.0 | 32.3 | 51.6 | 16.1 | 5.0 (4.1-8.0) | 11.3 (7.5-25.9) | 51 |
| Jeong 2023 | 62 | 68 (IQR, 59-74) | 61.3 | NR | 1.6 | 6.5 | 45.2 | 22.6 | 3.8 (IQR, 3.0-4.5) | 5.6 (IQR, 3.3-8.0) | NR |
| Jin 2023 | 20 | 58 (43–69) | 55.0 | 12.5 | 0.0 | 30.0 | 65.0 | 5.0 | 6.5 (4.2-8.8) | 12.3 (10.1-14.5) | 20 |
| Shi 2023 | 30 | 57 (25-73) | 63.0 | 23.5 | 3.3 | 76.7 | 13.3 | 3.3 | 10.2 (9.3-16.8) | 22.5 (15.6-29.3) | 56 |
| Wang 2023 | 49 | 62 (58-65) | 65.3 | 27.5 | 0.0 | 28.6 | 53.1 | 18.4 | NR | NR | 40 |
| Yoo 2023 | 159 | 65 (39-83) | 59.1 | 16.1 | 1.9 | 8.8 | 11.9 | 66.0 | 1.8 (1.7-1.8) | 7.6 (5.8-9.7) | 30 |
| Zhu&Li 2023 | 53 | 58 (51-66) | 62.3 | 13.7 | 5.7 | 47.2 | 41.5 | 5.7 | 8.6 (7.2-11.6) | 14.3 (11.3-NE) | 15 |
| Zhu&Xue 2023 | 57 | 59 (IQR, 51-64) | 64.9 | 15.1 | 3.5 | 40.4 | 47.4 | 8.8 | 9.3 (7.1-11.6) | 13.4 (10.0-NE) | 45 |

AE, adverse events; CI, confidence intervals; CR, complete remission; IQR, interquartile ranges; mPFS, median progression-free survival; mOS, median overall survival; PD, progressive disease; PR, partial response; NE, not estimable; NR, not reported; SD, stable disease
